# A Motor-Gradient and Clustering Model of the Centripetal Motility of MTOCs in Meiosis I of Mouse Oocytes

**DOI:** 10.1371/journal.pcbi.1005102

**Published:** 2016-10-05

**Authors:** Neha Khetan, Chaitanya A. Athale

**Affiliations:** Division of Biology, Indian Institute of Science Education and Research (IISER) Pune, Pune, Maharashtra, India; University of California San Diego, UNITED STATES

## Abstract

Asters nucleated by Microtubule (MT) organizing centers (MTOCs) converge on chromosomes during spindle assembly in mouse oocytes undergoing meiosis I. Time-lapse imaging suggests that this centripetal motion is driven by a biased ‘search-and-capture’ mechanism. Here, we develop a model of a random walk in a drift field to test the nature of the bias and the spatio-temporal dynamics of the search process. The model is used to optimize the spatial field of drift in simulations, by comparison to experimental motility statistics. In a second step, this optimized gradient is used to determine the location of immobilized dynein motors and MT polymerization parameters, since these are hypothesized to generate the gradient of forces needed to move MTOCs. We compare these scenarios to self-organized mechanisms by which asters have been hypothesized to find the cell-center- MT pushing at the cell-boundary and clustering motor complexes. By minimizing the error between simulation outputs and experiments, we find a model of “pulling” by a gradient of dynein motors alone can drive the centripetal motility. Interestingly, models of passive MT based “pushing” at the cortex, clustering by cross-linking motors and MT-dynamic instability gradients alone, by themselves do not result in the observed motility. The model predicts the sensitivity of the results to motor density and stall force, but not MTs per aster. A hybrid model combining a chromatin-centered immobilized dynein gradient, diffusible minus-end directed clustering motors and pushing at the cell cortex, is required to comprehensively explain the available data. The model makes experimentally testable predictions of a spatial bias and self-organized mechanisms by which MT asters can find the center of a large cell.

## Author Summary

Recent microscopy based time-lapse measurements of spindle assembly in the absence of pre-existing centrosomes, have improved our understanding of the principles of self-organization at a cellular scale. Mouse oocytes meiosis is one such example, where radial microtubule (MT) arrays, or asters, nucleated by MT organizing centers (MTOCs) centripetally converge on the central chromatin mass and reorganize to form a bipolar structure. This centripetal motility resembles a biased ‘search-and-capture’ process. Here we attempt to test whether such a bias is necessary and if so, to define the form and nature of the bias. We quantify the statistics of the experimentally measured motility and find MTOC transport involves both “pulling” from the center and “pushing” from the cortex. We hypothesize that the bias consists of a spatial gradient of forces driving this motility. To test this hypothesis, we develop a model of a random walk with drift in the mouse oocyte geometry. By optimizing the functional form of the drift field to experimental data, we then explore which minimal combination of molecular motors and dynamic instability parameters in such a gradient, could result in the generation of asymmetric forces. When minus-end directed dynein-like motors are localized in a gradient around chromatin, it reproduces the experimental spatio-temporal dynamics. By themselves, MT regulatory gradients, clustering motor complexes and cortical pushing fail to reproduce experimental statistics. A combination of the motor-gradient and clustering motors appears to explain all the available experimental statistics. Our findings suggest a possible functional role for previously observed enrichment of dynein around chromatin in mouse oocytes, and address how MTs asters find the center of a large cell, without the predominance of cortical interactions.

## Introduction

Spindle assembly in higher eukaryotic cells involves the self-organization of microtubules (MT) into a bipolar structure. During mitosis in animal cells, spindle poles are defined by a pair of centrosomes. However bipolar structures emerge even in the absence of centrosomes during meiosis in vertebrates as well as mitosis in plants. In such acentrosomal spindles, the poles self-organize by the dynamic interactions of MTs with molecular motors, regulatory factors and chromatin. While multiple components of this cellular-scale pattern forming system have been identified, the precise nature of the interactions between the components are still not completely understood.

The meiotic maturation of mouse oocytes is a well studied example of such an acentrosomal spindle assembly system. The first meiotic division is characterized by germinal vesicle breakdown (GVBD) [1], before and after which small aster-like fibrillar structures or microtubule organizing centers (MTOCs) are observed [2]. MTOCs which are nucleated both in the cytoplasmic and peri-nuclear spaces, both aggregate at the center to form a spindle by prometaphase I [3]. Such a convergence of radial MT arrays or asters was reported previously in *Xenopus* meiosis II oocytes [4]. Using cell-free *Xenopus* oocyte extracts, this convergence was shown to result from asymmetric centrosomal MT growth due to a gradient of RanGTP [5–7]- referred to as biased ‘search-and-capture’. However during meiosis I in mouse oocytes, experimental perturbation of RanGTP levels does not significantly affect spindle assembly [8, 9]. If RanGTP does not act as a guidance cue as reported previously [10], the nature of the directional cue and force generation remains to be understood.

The force required for MTOC convergence to the nuclear region is thought to originate from a combination of MTs, motors and anchorage points. Multiple mechanisms have been reported in the past to drive radial MT array transport in cells- (a) polymerization dependent pushing forces as seen during the centering of asters *in vitro* [11, 12], (b) cortical force-generator based pulling [13], (c) cortical motors which both depolymerize and pull [14], (d) cytoplasmic minus-ended motors which pull asters in a length-dependent manner [15], (e) cytoplasmic streaming by cargo transport driving aster movement [16, 17] and (f) acto-myosin contractility as seen in starfish oocytes [18]. Contact with the cell cortex can move asters when the relative MT lengths is comparable to the cell radius [19]. Both active and passive mechanisms drive the movement of centrosome nucleated asters. However most of the cortical pushing and pulling models are unlikely to affect long-range movement of MTOCs which have MT lengths ∼3 *μm* as compared to the cell-radius of ∼40 *μm*. Transport of asters by cytoplasmic streaming based on cargo transport by one large aster [17], is also unlikely to drive mouse meiosis I oocyte MTOCs due to their size and number (∼80 to 100), which will prevent a coherent and directed flow. Inhibition of acto-myosin contractility has also been shown to have no effect on the centripetal movement of mouse MTOCs [9]. While centrally-anchored MTOCs and cross-linking motors have also been proposed by Schuh et al. [9] to drive the MTOC motility, the movement continues even after nuclear envelope breakdown (NEBD). Thus for a complete theoretical understanding of the mechanism by which MTOCs converge in spindle assembly a mathematical model of the process is necessary to test multiple hypotheses that have been proposed.

Theoretical models have been used to probe the interactions of microtubules and motor complexes and are capable of reproducing *in vitro* self-organized patterns [20–22]. These simulations have been extended to understand the role of multiple components in spindle assembly such as antiparallel interactions [23], pole focussing by minus-end directed motors [24], gradients of stabilization [25] and intra-spindle nucleation and dynamic instability regulation [26]. In recent work, we have demonstrated the centripetal movement of centrosomal MT asters towards surface immobilized chromatin in *Xenopus* egg extracts can be modeled by a gradient of polymerization dynamics and uniform motor distribution [27]. This is comparable to a model of length-dependent pulling by motors to translocate MT asters during *C. elegans* embryogenesis [15]. However, neither of these models take into account the relatively shorter MTs seen in MTOC asters, and lack details specific to meiosis I. In search of common design principles in spindle assembly, theoretical modeling of the centripetal motility of MTOC arrays can be used to test the generality of previous results.

Here, we quantify the spatial trends in MTOC motility and find the random and directional components of motility depend on how far the MTOCs are from the cell center. The detailed quantitative analysis allows us to develop and test theoretical models of random walk with drift. Only a spatial gradient of drift can reproduce the experimental data. Such an optimized gradient is further used to model MT dynamic instability and motor distributions, to test the combination of mechanisms that can reproduce the experimental statistics of centripetal MTOC motility.

## Models

We have developed two kinds of models- phenomenological and mechanistic- to address both the general principles and specific mechanisms of the centripetal convergence of small MT asters, MTOCs. The models are:

1. 2D Random walk with drift (RWD) model
2. 2D MT-motor model

The outputs of both models are compared to 2D experimental motility measures of MTOCs from mouse oocyte meiosis I reported previously by Schuh and Ellenberg [9] The RWD model is used to optimize the functional form of the spatial drift field by comparison to experiment, while the MT-motor model is used to test molecular mechanisms which could generate the drift field. Mechanisms that have been previously hypothesized to drive asters to the center of cell involve MT-pushing and pulling [28, 29]. A combination of pulling and pushing mechanisms has been experimentally tested in sand-dollar eggs [30] and *C. elegans* embryos [16, 31] and pulling alone in *Xenopus* egg extracts [5, 27], while pushing has been seen in fibroblasts [32]. The MT-motor model is used to test whether the reported self-organized clustering of MTOCs in mouse oocytes [9] is sufficient to result in MTOC convergence, or whether additional mechanisms are necessary. These mechanisms are:

1. Self-organized mechanisms: not requiring explicit spatial localization
  - Cortical pushing alone
  - Clustering motors and cortical pushing
2. Spatial gradients: requiring explicit spatial localization
  - MT dynamic instability gradient
  - Dynein motor gradient
3. Hybrid model: combination of self-organized and gradient mechanisms

An optimization routine has been developed to compare the outputs of the ‘scenarios’, i.e. combinations of these mechanisms, to reproduce previous experimental reports of mouse meiotic MTOC motility and distribution [9, 33, 34].

**Fig 1:**
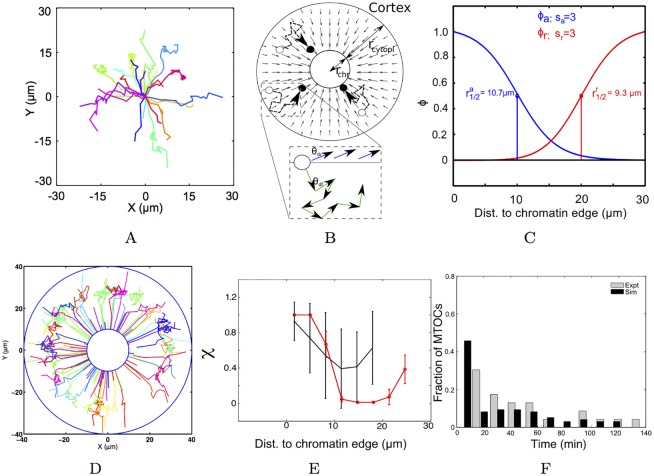
Optimizing the random walk with drift (RWD) gradient to experiment. (A) The experimentally measured 2D trajectories of MTOCs from mouse oocytes (from previous work [9], replotted based on raw data provided by the authors) are modeled by (B) a 2D RWD model with the concentric cytoplasmic and chromatin regions of radius *r*_*cyto*_ and *r*_*chr*_ respectively. The cytoplasmic motility of MTOCs from their initial positions (open circle) to final positions (closed circle) is driven by a field of drift (small arrows). *(inset)* The direction of motion is determined by the Brownian (*θ*_*df*_) and directed (*θ*_*dr*_) components. (C) The functional forms of the fields of attraction, $\phi_{a}\left(\vec{X}\right)$ (blue) and repulsion, $\phi_{r}\left(\vec{X}\right)$ (red), as a function of radial distance to chromatin (r) is determined by the respective *r*_1/2_ and *s* values. The optimized gradient results in (D) XY trajectories from simulation (colors indicate individual tracks), (E) directionality (*χ*) (mean ± s.d.) as a function of radial distance from simulation (red) compared to experiment (black) and (F) the frequency distribution of capture time (*t*_*c*_) from simulation (black) compared to experiment (grey).

The choice of a 2D model for a 3D spherical oocyte is determined by the need to compare simulated motility outputs with the only available experimental time-series dataset of MTOC motility in mouse oocyte meiosis I, which is 2D over time [9] (Fig 1A). This compatibility of dimensions is essential since some of the RWD model input parameters are obtained from fits to experiment and MT-motor model mechanisms are optimized based on their ability to reproduce experimental statistics. Additionally, the choice of dimensionality of spatial models is considered to be determined by the balance between the need to capture the behavior of the system sufficiently and the clarity of the model [35].

### Random walk in a drift field

The mouse oocyte is modeled in a 2D circular geometry of radius *r*_*cell*_, with concentric circular chromatin of radius *r*_*chr*_. The outer cytoplasmic region has a radius *r*_*cyto*_ and *r*_*cell*_ = *r*_*chr*_ + *r*_*cyto*_ (Fig 1B). MTOC asters are modeled as point particles, nucleated uniformly in the cytoplasmic space of the oocyte. The motion of the simulated particles is a mixture of random Brownian and directed centripetal motion, depending on the position of the MTOC in the oocyte (Fig 1B), based on the previously observed ‘stop and go’ nature of the motility [9]. In this model, MTOCs are transported to the cell center and ‘captured’ by the chromatin once they reach the central chromatin mass. The process resembles models of biased ‘search-and-capture’ used to describe spindle assembly [7, 36, 37]. Here, we use the model to define the spatial properties of the bias of attraction by comparing the simulation outputs to spatial trends in MTOC motility seen in experiment.

#### Velocity

The Brownian component of the motion results from radial MTs in an aster interacting with motors in all directions resulting in uniform forces, which fluctuate due to thermal noise and microtubule dynamics [27]. The equation of motion of the particle is given by its velocity ($\dot{X}$) and net angle (*θ*_*net*_). The magnitude of the velocity is described by:

$$\dot{X}=\left\{\begin{array}{cc} \sqrt{4\cdot D_{eff}/t}\text{,} & \mathrm{Brownian}\;\mathrm{component}\\ v_{eff}\text{,}\; & \mathrm{Directed}\;\mathrm{component} \end{array}\right.$$ (1)

where *D*_*eff*_ is the effective diffusion coefficient, *v*_*eff*_ is the effective velocity and t is time. The values of *D*_*eff*_ and *v*_*eff*_ are determined by fits to experimental data as described in the section on data analysis and listed in Table 1.

**Table 1:** RWD model parameters. Parameters are used from literature and fits to experimental data.

Parameter	Value	Reference
*Model geometry*		
Oocyte radius (*r*_*cell*_)	40 *μ*m	[9]
Chromatin radius (*r*_*chr*_)	10 *μ*m	[9]
*RWD model*		
Total simulation time (T)	8000 s	[8, 9]
Step time (*δ*t)	0.1 s	Optimized for numerical accuracy
No. of MTOCs (*N*_*p*_)	100	[8, 9]
Effective diffusion coefficient (*D*_*eff*_)	0.006 *μm*^2^/*s*	Fit to experiment (S1A and S1B Fig)
Effective velocity (*v*_*eff*_) of directed motion	0.008 *μm*/*s*	Fit to experiment (S1A and S1C Fig)

#### Direction

The net angle (*θ*_*net*_) of motion, which governs the direction of motion, is determined by:

$$\theta_{net}=\left\{\begin{array}{cc} \theta_{df}=rand\left(0,2\pi \right), & \mathrm{Brownian}\;\mathrm{component}\\ \theta_{dr}=f\left(\vec{XC}\right), & \mathrm{Directed}\;\mathrm{component} \end{array}\right.$$ (2)

where *θ*_*df*_ is the diffusive and *θ*_*dr*_ the directed angle. The angle *θ*_*df*_ can take random values uniformly distributed between 0 and 2*π*, while *θ*_*dr*_ is determined by the angle of the vector $\vec{XC}$ between the particle ($\vec{X}$) and the center ($\vec{C}$) of the cell (Fig 1B).

#### Drift field

Sub-cellular transport has been successfully modeled in previous work by combining Brownian motion and directed transport [38]. In our model, the Brownian and directed components are spatially determined. Here, two radially symmetric fields of drift, one repulsive and one attractive, were modeled to represent the effective forces acting on the MTOCs. The attractive field resulting in centrosomal aster convergence in *Xenopus* oocytes towards chromatin [5], was modeled by a sigmoid gradient [7, 27], A similar function was tested for the model of mouse oocytes. Since the attractive ($\phi_{a}\left(\vec{X}\right)$) and repulsive fields ($\phi_{r}\left(\vec{X}\right)$) determine the nature of motion, depending on the position of the particle in space, the net velocity of a particle (${\dot{X}}_{net}\left(x,y\right)$) is given by:

$${\dot{X}}_{net}\left(x,y\right)=\phi_{a}\cdot v_{eff}+\left(1-\phi_{a}\right)\cdot {\dot{X}}_{c}\left(x,y\right)$$ (3)

where ${\dot{X}}_{c}\left(x,y\right)$ is the velocity at the cell cortex determined by the weighted average of repulsion and Brownian motion. ${\dot{X}}_{c}$ is determined by:

$${\dot{X}}_{c}\left(x,y\right)=\phi_{r}\cdot v_{eff}+\left(1-\phi_{r}\right)\cdot \sqrt{4\cdot D_{eff}/t}$$ (4)

In this model, attraction originates from the central chromatin, since the removal of the nucleus in previous experiments resulted in MTOCs losing their directionality of motion [9, 39]. The repulsion from the cell cortex, is based on models of MT-aster pushing at the cell cortex as a mechanism of centering in multiple cell types [28, 29]. MTOCs undergo Brownian motion when neither attraction nor repulsion are acting on them. The net angle *θ*_*net*_(*X*) is the circular weighted average [40] of *θ*_*df*_ and $\theta_{dr}\left(\vec{XC}\right)$, weighted similarly to the velocity magnitude (Eqs 3 and 4).

The fields of $\phi_{a}\left(\vec{X}\right)$ and $\phi_{r}\left(\vec{X}\right)$ are modeled in 2D by radially symmetric functions dependent solely on the distance *r* from chromatin. A modified sigmoid gradient was tested, based on previous experimental measurements of long-range gradients acting on MTs around chromosomes in *Xenopus* spindle assembly [5–7] and theoretical models testing their form [25, 26] and their role in centrosome directional motility [27].

The field (*ϕ*) of either attraction (*ϕ*_*a*_) or repulsion (*ϕ*_*r*_) is determined by:

$$\phi =\frac{1}{1+e^{\left[\left(r-r_{\left(1/2\right)}\right)/s\right]}}$$ (5)

where *r* is the distance- for *ϕ*_*a*_, *r* = 0 at the chromatin edge and for *ϕ*_*r*_, *r* = 0 at the cell boundary. *r*_1/2_ is the distance at which the function takes the half-maximal value and 1/*s* is the steepness factor. While exponential gradients were also tested, they failed to reproduce the spatial trend in experimental data and are not shown here. Representative gradients of *ϕ*_*a*_ and *ϕ*_*r*_ are plotted as scaled values between 0 and 1 (Fig 1C).

### Mechano-chemical model of MT-motor interactions

A mechanistically detailed model was developed using Cytosim [41], a C++ Langevin dynamics simulation engine, by building on previously developed models of MT-mechanics [14, 42], polymerization kinetics [7, 25] and motor interactions [20–24, 26, 27, 43]. To test what minimal components will produce the observed centripetal motility of MTOC asters, a model of an MT stabilization gradient described previously [27] was developed by mapping the gradient shape optimized in the RWD model. This scenario was compared with scenarios where the gradient consisted of immobilized minus-ended molecular motors. These biased ‘search-and-capture’ scenarios were contrasted with self-organized scenarios which lacked any directional bias, i.e. diffusible motor-complexes and MTOC pushing from the cell boundary. The model is implemented in an oocyte cell geometry with MT polymerization dynamics and mechanics as well as discrete stochastic molecular motors that are either immobilized or diffusible.

#### Cell geometry

As before the oocyte was modeled in a 2D circular geometry with a concentric chromatin region. The chromatin region here is not treated as an absorber in this model, unlike in the RWD model.

#### MT polymerization dynamics and mechanics

MTs were modeled as discrete polymers undergoing dynamic instability- stochastic switching between growth and shrinkage phases- based on the four-parameter model [44, 45]. Since dynamic instability parameters of meiosis I mouse oocytes are not reported, values were taken from measurements made on centrosomal MTs in mitotic pig kidney cells, which have average MT lengths of 3.2 *μm* [46], very similar to the average length of MTs in mouse meiotic MTOCs (<*L* > ∼3 *μm*) [2, 3, 33]. The flexibility of the simulated MTs is defined by a combination of bending modulus (*κ*), typical cytoplasmic viscosity and thermal energy. The values of these parameters were taken from literature (Table 2), from the mouse oocytes or related systems, if mouse data was not available.

**Table 2:** MT-motor model parameters. The mecho-chemical parameters of motors were based on reported values for dynein, while motor densities were estimated. MT polymerization dynamics and mechanics is taken from literature, while cell geometry parameters are identical to Table 1.

Parameter	Value	Reference
Filament unit size	0.5 ⋅ 10^−6^ m	[23]
Time step	0.01 s	[23]
Total time	1200 s	[9]
Cytoplasmic viscosity (*η*)	0.05 *Pa* ⋅ *s*	[51]
Microtubule bending modulus (*κ*)	2 ⋅ 10^−23^ *N* ⋅ *m*^2^	[52]
Thermal energy scale (*k*_*B*_*T*)	4.1 ⋅ 10^−21^ *N* ⋅ *m*	-
*MTOC Parameters*		
Number of MTOCs per oocyte	80	[9, 34]
Number of MTs per MTOC (*N*_*MT*_)	20-120	[9, 34], estimate
Mean MT length (<*L*>)	3.2 *μm*	[3, 9, 34]
*MT polymerization dynamics*		
	*Cytoplasmic; Stabilized*	
Growth rate (v_*g*_)	0.178; 0.178 *μm*/*s*	[46], estimate
Shrinkage rate (v_*s*_)	0.205; 0.205 *μ*m/s	”
Catastrophe frequency (f_*cat*_)	0.075; 0.0397 1s^−1^	”
Rescue frequency (f_*res*_)	0.023; 0.0122 1s^−1^	”
*Motor parameters*		
Motor stiffness (*k*_*mot*_)	0.1 pN/nm	[53]
Motor (dynein) speed (*v*_*m*_)	2 *μ*m/s	[54–56]
Attachment rate (*r*_*attach*_)	12 s^−1^	[27]
Basal detachment rate ($r_{detach}^{{}^{\prime }}$)	1.5 s^−1^	[27]
Motor stall force (*f*_0_)	2 and 7 pN	[48, 55–57]
Attach distance (*d*_*attach*_)	0.02 *μm*/*s*	[27]
End detach rate ($r_{detach}^{end}$)	1 *s*^−1^	[27]
Diffusion constant of motor-complexes (*D*_*c*_)	20 *μm*^2^/*s*	[23]
Immobilized motors per oocyte ($N_{m}^{i}$)	10^2^ to 10^4^	Estimate
Diffusible motor complexes per oocyte ($N_{m}^{c}$)	10^3^ to 10^5^	Estimate

#### MTOCs

MTOCs were modeled as hollow circular structures of radius 0.2 *μm*, initialized randomly throughout the cytoplasmic region. Each MTOC had *N*_*MT*_ number of MTs uniformly distributed radially around the centrosome, forming an aster. MTOCs can move throughout the cell interior.

#### Motor mechanics

Molecular motors were modeled as described previously [27, 42] as discrete particles with properties taken from minus-end directed dynein-like motors moving at a velocity *v*_*m*_. Motors bind stochastically to MTs at an attachment rate *r*_*attach*_ only if the distance between them is less than a threshold distance of attachment (*d*_*attach*_). Detachment occurs at a rate *r*_*detach*_. Motors bound to MTs behave like Hookean springs with a spring constant (*k*_*mot*_), experiencing a extension force (*f*_*ex*_) parallel to the filament if attached to a motile filament. The rate of detachment increases with extension as: $r_{detach}=r_{detach}^{{}^{\prime }}\cdot e^{|f_{ex}|/f_{0}}$ (based on Kramers theory [47]), where $r_{detach}^{{}^{\prime }}$ is the constant basal detachment rate and *f*_0_ stall force. A separate $r_{detach}^{end}$ rate accounts for the rate at which motors detach from the end of a filament- here it is set to a value similar to $r_{detach}^{{}^{\prime }}$. As *f*_*ex*_ increases to values approaching *f*_0_, motor step sizes are expected to change, referred to as the ‘gear-like’ behavior of dynein [48]. This is modeled by a piece-wise approximation, as previously described [27, 49]. The parameters of motor mechanics, when available, are taken from experimental measurements of dynein (Table 2). In the absence of reported values estimates were used. Immobilized motors bind to microtubules and generate forces on the MTs causing their movement. Conversely, diffusible motor complexes with diffusion coefficient *D*_*c*_, are modeled as crosslinkers [23], which can bind two different microtubules. Since aster have minus-ends at the center, cross-linking minus-end directed motility of the motors results in coalescence of the MTOCs.

#### Aster motility

MTOC asters move due to a net force calculated by resolving multiple forces acting on the MTs (using a finite difference scheme to solve the Langevin equation of motion in Cytosim [42]) which are: (a) *f*_*bend*_, arising due to bending of growing MT filaments contact the rigid cell boundary and scaled by the bending modulus of MTs (*κ*) [11, 12, 50]; (b) $f_{ex}^{i}$, the extension force generated when immobilized motors bound to MTs walk on the filament and are stretched by length *x*, experience a restoring force $f_{ex}^{i}=k_{mot}\cdot x$, resulting in filament motion [42]; (c) $f_{ex}^{c}$, the motor-complex of diffusible tetrameric motors have two binding sites separated by a spring of stiffness *k*_*mot*_. Complexes that are simultaneously bound to two MTs, undergo stretching. The restoring spring force brings the two MTs close and results in clustering [20, 22, 23]; (d) *f*_*diff*_, the diffusive force is a random normally distributed force [42]; and (e) *f*_*drag*_, the drag force acting on the MTs resulting from the translational and rotational drag forces on the individual points representing the filament [42] based on the cytoplasmic viscosity (*η*). Parameters are reported in Table 2.

### Methods

#### Data analysis

Experimental 2D trajectories of multiple MTOCs in mouse oocytes meiosis I described previously by Schuh and Ellenberg [9] with Δ*t* ≈ 3 to 4 minutes) ranging between pre- and post-NEBD stages (data kindly shared by M. Schuh) were normalized by origin shift to the respective end-points. Thus, multiple experimental trajectories could be compared. For comparison with experiment, simulated trajectories were down-sampled to a time-interval comparable to experiment (Δ*t* = 3.5 minutes), and the following measures of motility were evaluated from both experimental and simulated data:

*Motility statistics:* The instantaneous velocity *v* = *δL*/*δt* and directionality or tortuosity (*χ*) was estimated as *χ* = *d*_*net*_/*L*. A value *χ* = 1 indicates directional motion, while *χ* ∼ 0 indicates a random (tortuous) path. The time taken by MTOCs from their time of nucleation to arrive at the edge of the central chromatin region is used to calculate a ‘capture time’ from both experiments and simulations. The mean square displacement (msd) of experimental and simulated trajectories was estimated by:

$$\langle \Delta r^{2}\rangle =\langle {\left[r(t)-r(t+\delta t)\right]}^{2}\rangle$$ (6)

where *r* is the displacement, *t* is the time point, *δt* is the time increment. A sliding window of *δt* from the smallest simulated time step to 3/4th of the trajectory length was used [27]. This empirically determined cut-off reduces artifacts arising from the fact that the number of steps of time-length greater than this threshold are extremely small [58, 59].

In order to estimate input parameters for the RWD model (Eq 1), the msd profiles of experimentally measured MTOCs were fit to the model of diffusion and transport:

$$<r^{2}>=4\cdot D_{eff}\cdot \delta t+{\left(v_{eff}\cdot \delta t\right)}^{2}$$ (7)

On the other hand, the msd output from the motor-MT interaction model (Models section) were quantified by fitting to an ‘anomalous diffusion’ model:

$$<r^{2}>=4\cdot D^{{}^{\prime }}\cdot t^{\alpha }$$ (8)

to estimate the apparent diffusion coefficient (*D*′) and the anomaly parameter (*α*) as described previously [27] (S1 Text). All data fitting was performed using the trust region reflective least square fitting algorithm implemented in the *Optimization Toolbox* of MATLAB (Mathworks Inc, USA).

#### Simulations

The RWD simulation time steps were optimized for numerical stability, by simulating a normal random walk (*α* = 1) with no boundary conditions and minimizing the error in input and fit diffusion coefficient (*D*). Simulations with typically 100 particles were run for 8 ⋅ 10^3^ seconds and took ∼20 minutes. The explicit MT-motor model (Cytosim) with 80 MTOCs was run for 1.2 ⋅ 10^3^ seconds, typically requiring ∼8 hours on a 12 core Intel Xeon machine with 16 GB RAM.

#### Model optimization to experiment

The parameters of the model were optimized by a rank minimization method based on a modified weighted root mean square error (*ϵ*) of simulations as compared to experiment. Only one published dataset by Schuh and Ellenberg [9], has reported detailed XY over time trajectories of MTOC centering motility during mouse oocyte maturation, as a result of which quantitative model optimization was performed on this dataset (kindly provided by M. Schuh). The error (*ϵ*(*k*)) for a parameter set *k* was estimated between the *i*^*th*^ experimentally measured (*e*_*i*_) and simulated (*s*_*i*_) values by:

$$\epsilon \left(k\right)=\sqrt{\frac{1}{n}\cdot \sum_{i=1}^{n}w_{i}\cdot {\left(e_{i}-s_{i}\left(k\right)\right)}^{2}}$$ (9)

The weight (*w*_*i*_) was determined based on whether the simulated value was within one *σ* of the average experimental measure or outside that range:

$$w_{i}=\left\{\begin{array}{cc} n_{i}/n_{max} & s_{i}\forall \left(e_{i}\pm sd\right)\\ w_{m} & s_{i}\;∼\left(e_{i}\pm sd\right) \end{array}\right.$$ (10)

where *w*_*m*_ = 2, *n*_*i*_ is the number of experimental data-points for the *i*^*th*^ value and *n*_*max*_ is the maximal number of data-points in experiment. This weighting scheme allows us account for the increased uncertainty in experimental measurements with fewer data points. The choice of *w*_*m*_ was set empirically to 2 to ensure a high penalty (since *n*_*i*_/*n*_*max*_ ≤ 1). For each parameter set the *ϵ* of multiple variables (*v*) were ranked (*R*_*v*_(*k*)). The directionality (*χ*) distribution with distance (*r*) and frequency distribution of capture times (*t*_*c*_) were used to calculate errors. The sum rank, *R*_*s*_ = *Σ*_*v*_ *R*_*v*_(*k*) was minimized to obtain a parameter set, referred to as optimal.

## Results

### RWD simulated MTOC motility compared to experiment

The experimental trajectories of MTOCs show a distinct centripetal motion as seen in the time-projected trajectories (Fig 1A). The input parameters for diffusive and directed motion in the RWD model (Fig 1B) were obtained from fitting Eq 7 to experimental msd profiles (S1A Fig) and obtaining the mean *D*_*eff*_ (S1B Fig) and *v*_*eff*_ (S1C Fig). As a result, the nature of the centripetal motility depends solely on the parameters determining the field of drift. We optimize the parameters that determine the shapes of both attractive (*ϕ*_*a*_) and repulsive (*ϕ*_*r*_) fields (Fig 1C) by scanning 600 possible combinations, and minimizing the sum rank of errors (S2A Fig) obtained from the ranked errors (*ϵ*) between simulated and experimental values of *χ* and *t*_*c*_. Our minimization scheme identifies the optimal gradient to be a long range attractive gradient (*r*_1/2_ = 10 *μm* and *s* = 1) and a short range repulsive gradient from the cell boundary (*r*_1/2_ = 0 *μm* and *s* = 2) based on directionality and capture time. While the error (*ϵ*) minima do not coincide (in terms of gradient parameters) between *χ* (S2B Fig) and *t*_*c*_ alone (S2C Fig), our sum-rank scheme gives us a global optimum. The outputs of such a gradient result in XY trajectories which are directed inwards at the cell boundary, random in the mid-zone and directed closer to chromatin (Fig 1D), qualitatively comparable to experiment (Fig 1A). Quantitative comparisons between experimental and simulated *χ* profile reflects this trend in motility- particles at the cell boundary and near chromatin are more directed, than those in the mid-zone (Fig 1E). The simulated capture time distribution also matches with experiment (Fig 1F). In order to understand the mechanism underlying the experimental motility observed, trajectories were further analyzed for their time-dependence.

### Pushing and pulling profiles in MTOCs transport

**Fig 2:**
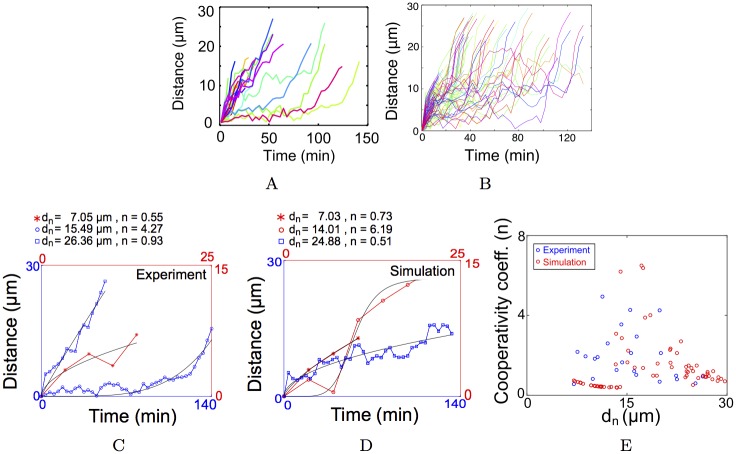
Distance travelled by MTOCs in experiment and simulation. The distance travelled from the site of nucleation is plotted as a function of time from (A) experiment and (B) optimized RWD gradient model. Colors indicate individual tracks. Representative fits (black line) to Eq 11 of data from (C) experiment and (D) simulation. Blue and red indicate the axes which scale the respective distance-time graphs. *d*_*n*_ is the distance from chromatin where the MTOC is nucleated and *n* is the cooperativity coefficient from the fit. (E) The fit values of *n* from experiment (blue) and simulation (red) are plotted as a function of *d*_*n*_. The goodness of fit *R*^2^ > 0.7 for all plots.

The experimentally measured MTOCs have heterogeneous distance-time profiles, with some MTOCs moving rapidly in < 40 min, while others undergo a delayed (> 40 min) inward movement (Fig 2A). The optimized RWD model profiles qualitatively match those from experiment. In previous work, distance-time plots with a sigmoid profile have been interpreted to mean pulling forces are at work, while a parabolic has been interpreted to mean pushing is at play [15]. Here, we use a fit function with three parameters, *n*: a measure of the shape of the profile (*n* > 1: sigmoid and *n* ≤ 1: parabolic), *T*_*half*_: the time at which the distance travelled is half-maximal, and *d*_*max*_: the maximal distance travelled, as follows:

$$d\left(t\right)=d_{max}\cdot t^{n}/\left(T_{half}^{n}+t^{n}\right)$$ (11)

The experimental and RWD simulation distance-time plots were fit to obtain *d*_*max*_, *T*_*half*_ and *n*. A value of *n* > 1 is taken to indicate pulling, while *n* ≤ 1 is taken to indicate pushing. Representative data from experiment (Fig 2C) chosen based on their nucleation position close to chromatin (*d*_*n*_ = 7.05 *μm*), mid-way in the cytoplasm (*d*_*n*_ = 15.49 *μm*) and close to the cell membrane (*d*_*n*_ = 26.36 *μm*), show an apparent pattern in the *n* values- high in the mid-zone and low close to chromatin and near the cell boundary. Representative plots from the optimized RWD simulations (Fig 2D) with *d*_*n*_ = 7.03 *μm*, *d*_*n*_ = 14.01 *μm* and *d*_*n*_ = 24.88 *μm* show a similar trend in the values of *n*. When the *n* from all experimental data (S3 Fig) is compared to simulated fits for different positions of nucleation, a qualitative match is observed (Fig 2E). The *n* value from simulations is higher in the mid-cell region as compared to near chromatin or at the cell boundary. This can be understood in terms of the sharp transition in the attractive gradient of drift (*ϕ*_*a*_) at *r* ≈ 15 *μm*, resembling a ‘pulling’ process. The phenomenological model does not allow an interpretation of the origin of pushing or pulling forces. We therefore proceeded to add detailed molecular-motor and MT polymerization dynamics to the model to make experimentally testable predictions about the system.

### Mechanistic models of MTOC centering: Gradient and Self-Organized Models

The RWD model predicts two drift fields- attractive from the center and repulsive from the cell boundary- are required to reproduce experimental statistics. Here, we proceed to test molecular mechanisms which combine molecular motors, MT-dynamics and mechanics of MTs and membrane interactions, with the aim of developing a mechano-chemical understanding of the effective drift fields. We categorize these mechanisms into two types:

1. Self-organized
2. Gradient-based

We then systematically evaluate plausible mechanisms and evaluate them for their ability to result in MTOCs finding the center of the oocyte within an experimentally observed time-scale (∼20 min).

#### (i) Self-organized mechanisms

**Fig 3:**
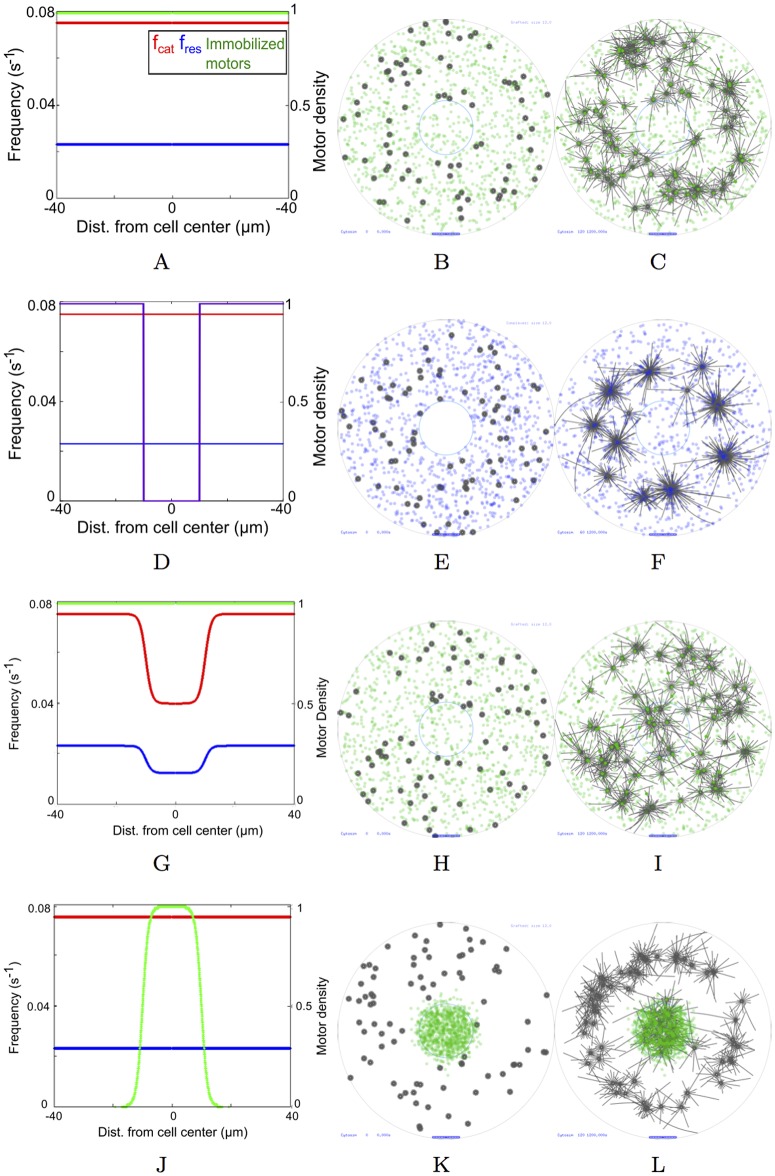
MT-motor model scenarios. (*Left column*) The distributions of *f*_*cat*_ (red), *f*_*res*_ (blue) and motor density of immobilized minus-ended motors (green) and (at t = 0) diffusible minus-ended motor complexes (purple) are plotted as a function of radial distance from the oocyte center. (*Middle column*) Simulation output at t = 0 minutes and (*Right column*) t = 20 minutes with MTOCs (grey) and a concentric nucleus (blue circle) with immobilized minus-ended motors (green dots) and diffusible tetrameric motor complexes (purple dots). Scenarios simulated: (A, B, C) Uniform distribution of *f*_*cat*_ and *f*_*res*_ and surface-immobilized motors $N_{m}^{i}=10^{3}$ motors/oocyte, *f*_0_ = 7 pN (green dots). (D, E, F) Diffusible minus-end directed motor complexes $N_{m}^{c}=10^{3}$ motors/oocyte, *f*_0_ = 7 pN (purple dots) and uniform *f*_*cat*_ and *f*_*res*_. (G, H, I) Gradients of *f*_*cat*_ and *f*_*res*_ with uniform immobilized minus-ended motors $N_{m}^{i}=10^{3}$ motors/oocyte, *f*_0_ = 7 pN (green dots). (J, K, L) Gradient model of motor density with $N_{m}^{i}=10^{3}$ motors/oocyte, *f*_0_ = 7 pN and uniform *f*_*cat*_ and *f*_*res*_ values. Scalebar 10 *μm*.

*(a) Cortical pushing.* In somatic cells the MT-asters find the center of a cell entirely based on pushing interactions at the cell cortex [32]. We test if pushing from the cell boundary alone, could result in MTOC centering, while maintaining uniform MT polymerization and immobilized dynein-like motors (Fig 3A). MTOCs at the start of the simulation are localized randomly throughout the oocyte (Fig 3B). However even after 20 minutes, asters did not move centripetally (Fig 3C, S1 Video).

*(b) Clustering motors and cortical pushing.* Based on the self-organized centripetal motility of MTOCs observed experimentally in mouse oocytes, clustering motor complexes had been proposed to be the major driver of MTOC motility to the center of the cell [9]. As a result, we modeled diffusible minus-end directed (dynein-like) motor complexes uniformly in the cytoplasmic region (Fig 3D and 3E). These complexes couple two nearby MT filaments and as a result of minus ended-motility, result in clustering of the asters (since asters have their minus-ends at the MTOC center). In this scenario, dynamic instability parameters were uniform and no immobilized motors were modeled. The complexes diffuse through the whole cell including the chromatin region within ≈ 30 s, mimicking start of NEBD (S2 Video). However even after 20 min of simulations, only a small fraction of the simulated MTOCs are inside the chromatin region (Fig 3F, Table 3).

**Table 3:** Percentage of MTOCs captured. After 20 min simulation time the proportion of MTOCs captured were evaluated for the different scenarios in the mechanistic models and compared to experimental values on a similar time-scale. Values are estimated from 10 runs of a typical simulation with 80 MTOCs each.

Model	Motor type	Localization	*f*_0_ (pN)	*N*_*m*_	% captured
*Self-organized scenarios*					
No gradient	Minus-end directed	Uniform, immobilized	2	10^3^	0.5
			7	10^3^	3.125
Clustering	Minus-end directed, tetrameric	Uniform, diffusible	2	10^4^	0.375
			7	10^4^	0
*Gradient scenarios*					
Stabilization gradient	Minus-end directed	Uniform, immobilized	2	10^3^	2.375
			7	10^3^	8
Motor gradient	Minus-end directed	Gradient, immobilized	2	10^3^	18.5
			7	10^3^	32
Experiment					30.43

#### (ii) Gradient-based mechanisms

*(a) MT dynamic instability gradient.* Based on evidence of a gradient of dynamic instability in multiple cell types [5, 7, 10, 36], we tested a gradient of *f*_*cat*_ and *f*_*res*_ as a mechanism for centering of asters, while maintaining a uniform surface-immobilized motor distribution (Fig 3G) and random MTOC nucleation (Fig 3H). However, this too did not result in a perceptible increase in accumulation of MTOCs at the chromatin center at the end of 20 minutes (Fig 3I, Table 3, S3 Video).

*(b) Dynein motor gradient.* It was only when motors were localized in a chromatin-centered sigmoid gradient and dynamic instability parameters were homogeneous (Fig 3J), did the randomly nucleated MTOCs (Fig 3K) dramatically converge to the center (Fig 3L, S4 Video).

Thus, a minimal model of asymmetric pulling forces resulting from a gradient of immobilized motors can move MTOCs to the center of the cell in a time-scale comparable to experiments (∼20 min). However, a quantitative comparison of the simulation statistics with experiment is necessary to understand the critical parameters in this model.

### Model sensitivity to stall-force and density of motors and MTs per aster

**Fig 4:**
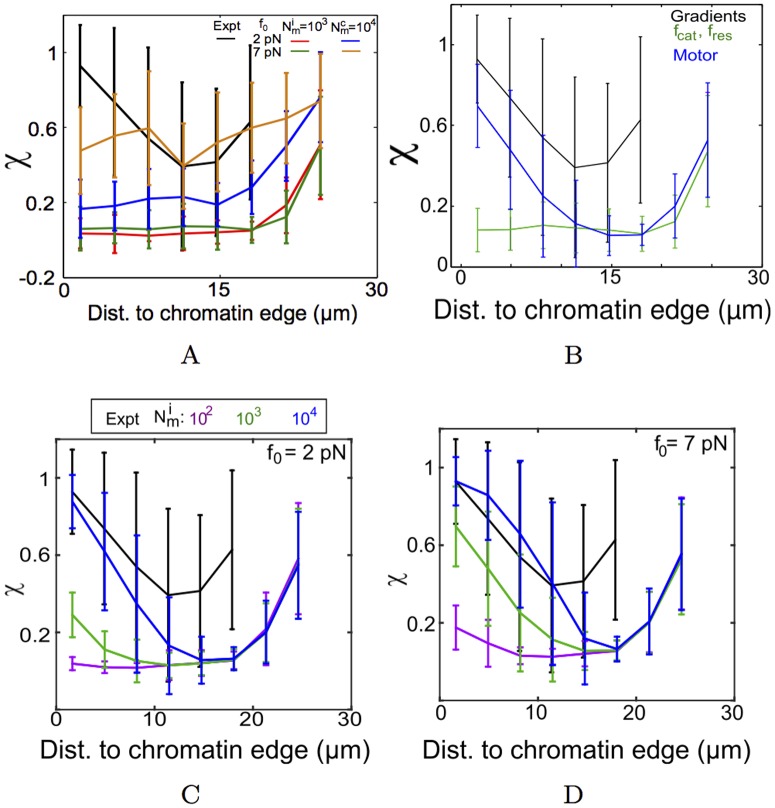
Directionality of simulated MTOCs compared to experiment. The distance dependence of binned directionality (*χ*) from experiment (black line) is compared to multiple simulated scenarios. (A) Uniformly distributed motors which are either immobilized weak (*f*_0_ = 2 pN) (red) or strong (*f*_0_ = 7 pN) (green) dynein-like motors with $N_{m}^{i}=10^{3}$ motors/oocyte are compared to tetrameric diffusible minus-end directed dynein like motor complexes with *f*_0_ = 2 pN (blue) and *f*_0_ = 7 pN (orange), with $N_{m}^{c}=10^{4}$ motors/oocyte. (B) The *χ* resulting from a gradient of *f*_*cat*_ and *f*_*res*_ (green) is compared to a gradient of the immobilized motors ($N_{m}^{i}=10^{3}$ motors/oocyte, *f*_0_ = 7 pN) (blue). In a gradient of immobilized motors with (C) *f*_0_ = 2 pN and (D) *f*_0_ = 7 pN the radial distribution of *χ* is plotted for increasing motor densities: $N_{m}^{i}=10^{2}$ (purple), 10^3^ (green) and 10^4^ motors/oocyte (blue). All values of *χ* are mean ± s.d. Simulations were averaged from 10 runs with 80 MTOCs each.

In the previous section, qualitatively, self-organized mechanisms of MTOC centering could not drive centripetal motility. The spatially binned directionality (*χ*) of simulated MTOCs in the absence of any gradient further quantifies this (Fig 4A). Neither tetrameric motor complexes, nor uniform surface immobilized motors and pushing at the cell boundary result in a trend in *χ* comparable to experiment. Cross-linking by motor complexes of different stall forces (*f*_0_ = 2 and 7 pN) and densities ($N_{m}^{c}=10^{3}$ and 10^4^ motors/oocyte) were tested and higher stall forces with high densities result in high values of *χ* throughout the cell (> 0.5). A directional bias in the form of a field of *f*_*cat*_ and *f*_*res*_ (based on Eq 5) resulting in asymmetric MT lengths, also fail to reproduce the directionality trends (Fig 4B). Confirming our qualitative observations from the simulation visualization, only a gradient of motors (*f*_0_ = 7 pN, $N_{m}^{i}=10^{3}$) can reproduce most of trend in directionality as a function of distance (Fig 4C and 4D).

In the absence of experimental estimates of the number of motors and their stall forces from meiotic mouse oocytes, we explore two extreme values of *f*_0_ (2 and 7 pN) reported in literature for dynein and scan $N_{m}^{i}$ over thee orders of magnitude. We find high density ($N_{m}^{i}=10^{4}$ motors/oocyte) of weak motors (*f*_0_ = 2 pN) (Fig 4C) and a lower density ($N_{m}^{i}=10^{3}$ motors/oocyte) of strong motors (*f*_0_ = 7 pN) (Fig 4D), can both reproduce experimental profiles of directionality. The proportion of MTOCs captured at the chromatin boundary was evaluated by following the distance of MTOC centers as they entered the chromatin mass. Strikingly the proportion of MTOCs captured in the first 20 minutes of simulation from the motor gradient with immobilized motors of *f*_0_ = 7 pN and $N_{m}^{i}=10^{3}$ most closely matched experiment (Table 3). However, the mean velocity values from simulations were insensitive to either *f*_0_ = 2 or 7 pN and motor densities over a range *N*_*m*_ = 10^2^ to 10^4^ motors/oocyte (Table 4).

**Table 4:** MTOC velocity in a motor gradient. The mean instantaneous velocity (<*v*> ± s.d.) with increasing motor density was calculated from 20 min simulation time and compared to experiment.

Total no. of motors ($N_{m}^{i}$)	<*v*> (10^−3^ *μm*/*s*)	
	f_0_ = 2 pN	f_0_ = 7 pN
10^2^	4.3 ± 6.3	4.8 ± 4.5
10^3^	4.8 ± 4.6	5.4 ± 5.7
10^4^	5.4 ± 5.7	5.4 ± 5.7
Experiment	8.7 ± 6.0	

**Fig 5:**
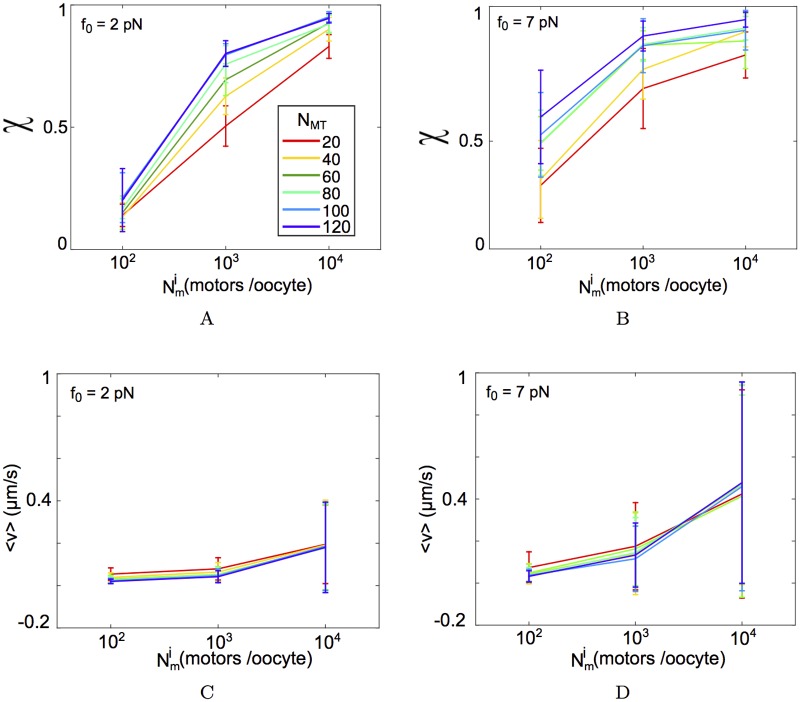
Effect of varying MTs per aster and motor density. MTOCs in the optimized motor gradient nucleated at *d*_*n*_ = 15 *μm* from the chromatin edge, were simulated for increasing MTs per aster (*N*_*MT*_): 20, 40, 60, 80, 100 and 120 (colors). (A, B) The directionality *χ* and (C, D) mean velocity (〈*v*〉) are plotted for *f*_0_ = 2 pN and, *f*_0_ = 7 pN for motor densities ranging between $N_{m}^{i}=10^{2}$ to 10^4^ motors/oocyte (x-axis). All values are mean ± s.d. from 10 asters.

Additionally to test how the motility was affected by the total number of MTs per aster in the scenario of a motor gradient, we examined the *χ* and <*v*> of simulated MTOCs which were initialized at a distance of 15 *μm* from the chromatin edge. As a result we expect these asters to experience the maximal force asymmetry, as they are at the lower end of the motor gradient. We find for increasing motor densities ($N_{m}^{i}$) directionality *χ* continues to increase, but increasing *N*_*MT*_ per aster appears to rapidly saturate the *χ* value for any given $N_{m}^{i}$ value for both *f*_0_ = 2 and 7 pN (Fig 5A and 5B). Increasing $N_{m}^{i}$ leads to a marginal increase in the mean velocity (<*v*>) for a fixed *N*_*MT*_ value. However increasing the value of MTs per aster, to our surprise, does not affect <*v*> (Fig 5C and 5D). We interpret this to be the result of the uniform radial distribution of MTs in the aster and a tug-of-war arising from it.

In order to further understand the role of motor-density changes and their effect on MTOC motility, we evaluate the random walk statistics of the motility and compare it to experiment.

### A hybrid model of a motor-gradient and self-organized clustering enhances MTOC centering

**Fig 6:**
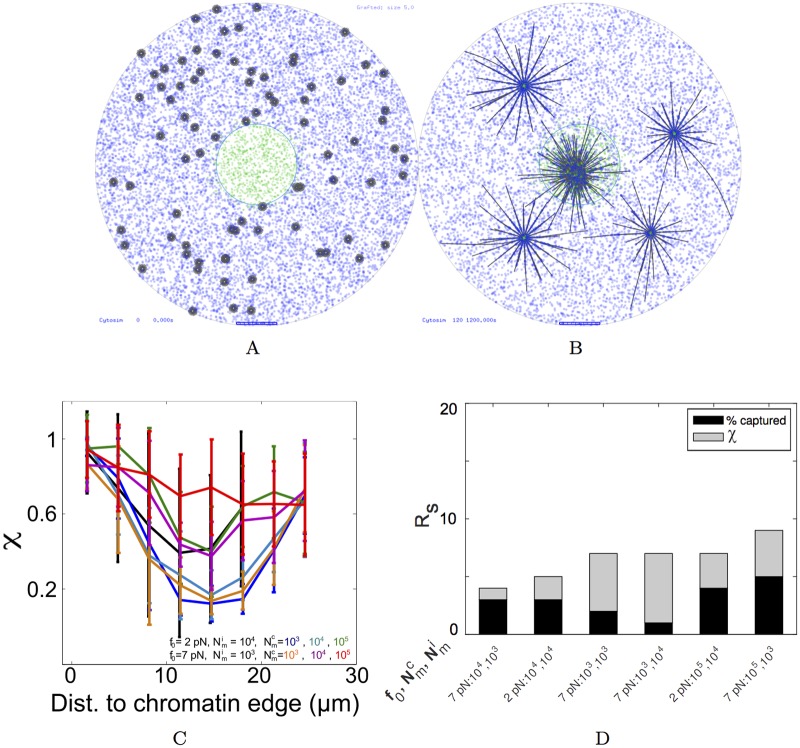
Combining immobilized and clustering motors. Simulation outputs of MTOCs (grey) motility in the presence of a combination of diffusible tetrameric minus-ended motor complexes ($N_{m}^{c}=10^{4}$, *f*_0_ = 7 pN) with chromatin-centered gradient of immobilized motors ($N_{m}^{i}=10^{3}$ motors/oocyte, *f*_0_ = 7 pN) at (A) t = 0 and (B) t = 20 minutes. (C) The *χ* values of aster movement resulting from increasing densities of weak (*f*_0_ = 2 pN) and strong (*f*_0_ = 7 pN) clustering motors ($N_{m}^{c}=10^{3}$ to 10^5^ motors/oocyte) in the presence of a gradient of immobilized motors with the same *f*_0_ values ($N_{m}^{i}=10^{4}$ for *f*_0_ = 2 pN and $N_{m}^{i}=10^{3}$ for *f*_0_ = 7 pN). (D) The sum rank (*R*_*s*_) based on the sum of square errors (*ϵ*) from *χ* distributions (grey) and % MTOCs captured in 20 minutes (black) from experiment are plotted in an ascending order, with the parameters of the density of immobilized motors ($N_{m}^{i}$) and diffusible motor complexes ($N_{m}^{c}$) plotted, assuming the *f*_0_ is constant for both.

Simulations of multiple gradient forms, motor types and densities in this work suggest a motor gradient as a minimal model to understand the centering motion of MTOCs in mouse meiosis I oocytes. However, we were unable to reproduce the experimentally observed *χ* ∼ 0.5, when MTOCs were 10-20 *μm* from the chromatin edge. Interestingly, the presence of the self-organized clustering motors (*f*_0_ = 7 pN, $N_{m}^{c}=10^{4}$ motors/oocyte) alone resulted in simulated *χ* values that matched experiment, in the radial distance between 10 and 20 *μm* (Fig 4A), due to MTOC aggregation. We hypothesized that a hybrid mechanism combining an immobilized motor-gradient with diffusible clustering motor-complexes might reproduce the complete experimental distance-dependent directionality profile. Visually the MTOCs appear to find the center more efficiently (Fig 6A and 6B). Here 10^4^ clustering motors per oocyte of stall force 2 pN were combined with 10^4^ immobilized motors per oocyte and stall force 2 pN. A systematic screen of the effect of increasing tetrameric complex density while keeping the density of immobilized of motors constant was evaluated in terms of the measure of directionality. The radial distance profile of *χ* increases in the mid-range when clustering complex density is increased from 10^3^ to 10^5^ motors/oocyte (Fig 6C). Either ‘weak’ diffusible dynein-like complexes (*f*_0_ = 2 pN) with a high density ($N_{m}^{c}10^{5}$) or ‘strong’ motors (*f*_0_ = 7 pN) with a lower density ($N_{m}^{c}=10^{4}$ motors/oocyte), both result in *χ* matching the experimental values in the mid-zone of the cell (10-20 *μm*) (Fig 6C and 6D). This result suggests that while clustering alone cannot center MTOCs in oocytes, it improves the fit to experiment.

Thus we believe our model reproduces both the qualitative and quantitative nature of the MTOC centering motility and provides novel insights into the sensitivity of this model. It demonstrates how a combination of directional cues and self-organized clustering can center small MT asters in a large cell such as an oocyte.

## Discussion

Meiotic spindle assembly in mammalian cells in the absence of centrosomes involves the nucleation of MTOCs in cytoplasm and their coalescence and sorting around chromosomes, resulting in bipolar spindle assembly. The nucleation of MTOCs in cytoplasm and the centripetal motility of small radial MT asters has been previously observed during the first meiotic division in mouse oocytes [3]. Similar convergence was also observed in *Drosophila* oocytes [60] and quantitative analysis and model calculations were used to infer that directed transport for the MTOCs was essential for the coalescence of MTOCs [61]. In *Drosophila* the unconventional kinesin Ncd (minus-end directed) was implicated to play a role in this inward motility [62]. In mouse oocyte MTOC coalescence, cytoplasmic dyneins or comparable minus-end directed motor have been implicated in force generation for the centripetal movement [9]. Here, we analyze experimental data from the early stages of the meiotic maturation of mouse oocytes. The experimental analysis is used to constrain a field of spatially inhomogeneous drift in a random walk. The optimized model of drift is used to model gradients of the motor dynein and MT dynamic instability. By comparing the simulation outputs with experiments, we arrive at a minimal model of a gradient of motors, essential to reproduce the experimentally observed statistics.

The experimental statistics of the MTOC motility from mouse oocytes are mostly representative of post-NEBD dynamics, allowing us make the simplifying assumption that the nuclear envelope plays no explicit role in the process. The movement of these radial MT arrays appears visually to have both an effectively diffusive and a transport component (Fig 1A). The frequency distribution of velocity from experiment is long tailed and fit to a lognormal function (S4 Fig), suggesting anomalous super-diffusive transport. While the motility had been previously described as ‘stop-and-go’ [9], we find little evidence of ‘stop’ or pause events in the motility. Our quantification of MTOC motility in cells, demonstrates the motility is qualitatively comparable to previous estimates of centrosomal aster movement observed in *C. elegans* fertilization [15], MTOCs in *Drosophila* oocyte meiosis I [61] and centrosomal asters in *Xenopus* meiotic extracts [5, 27]. This suggests a common theme underlying the transport of radial MT arrays in meiotic spindle assembly.

The distance travelled or displacement from the start-point plotted over time of MT arrays have been used previously [15] to distinguish between “pulling” and “pushing” modes of motility of centrosomal MT arrays during pronuclear migration. In the case of mouse meiotic MTOCs, these plots also help to distinctly separate trajectories into two sub-populations- those which show an initial rapid rise followed by capture (< 40 min), while others do not move much for a long time (> 40 min) and after this delay are captured at chromatin (Fig 2A). We further improve on the work of Kimura et al. (2005) [15] by fitting the data with a saturation model with cooperativity (Eq 11), and use it quantitatively to distinguish between pushing (*n* ∼ 1) and pulling (*n* > 1) mechanisms (Fig 2C). Every trajectory from experiment was fit to obtain a profile shape (sigmoid or parabolic) term *n* (S3 Fig) and comparing *n* from simulation and experiment shows a pushing mode of transport (*n* ∼ 1) close to chromatin and the cell boundary, while those in the mid-range are pulled (*n* > 1) (Fig 2E). The distance travelled plots from the mechanistic motor-gradient model with MT asters sorted by nucleation distance demonstrate an increase after a short delay near chromatin (*d*_*n*_ = 0 to 10 *μm*) due to pulling (S5A Fig). Those in the intermediate range (*d*_*n*_ = 10 to 20 *μm*) have a longer delay and then appear to be pulled (S5B Fig). Close to the cell-boundary (*d*_*n*_ > 20 *μm*) they increase rapidly and then saturate, due to MT-pushing at the membrane (S5C Fig). Thus a model of a gradient of chromatin-centered motors and a rigid cortex can reproduce the qualitative differences observed in the distance-time profiles of MTOC transport. However in these experiments, the absence of clear ‘pulling’ in experiment (*n* > 1) near chromatin might have been missed, since the MTOC identity is lost as it nears the chromatin. While cell cortex-based pushing mechanisms have been demonstrated to generate centripetal movement [12, 50], the nature and localization of minus-end directed pulling motors in the oocyte remains to be determined. Recent evidence of from mouse oocytes during MTOC fragmentation [63] and meiotic maturation [34] suggest dynein anchored at the nuclear envelope might influence both processes. A careful study of dynein localization dynamics in this and related systems, could by used to test our model prediction.

A ‘tug-of-war’ in the transport of anti-parallel MTs moving on a surface coated with motors arises from the action of the same species of motor acting against each other, with small asymmetries in length, amplifying the velocity of transport [64]. Two-fold length asymmetries (10 to 20 *μm*) have been previously observed in centrosomal asters [5, 7] and simulations of such asymmetric asters on sheets of dynein motors resulted in aster transport towards chromosomes [27]. However, the mouse MTOC radius is in the range of 2 to 3 *μm* and no appreciable asymmetry of lengths has been reported [9]. In this work, neither homogeneous motor distributions (S1 Video), nor tetrameric motor-complexes (S2 Video), nor a dynamic instability gradient (S3 Video) result in convergence to chromatin in the ∼20 min time scale seen in experiment. Taken together, a gradient of motors is necessary in a minimal model with a mouse oocyte geometry for MTOCs to converge to the chromatin center (S4 and S5 Videos). This suggests aster motility in meiosis I of mouse oocytes differs from that in meiosis II of *Xenopus* oocytes. In the latter, length asymmetry can result in directional transport, however in mouse oocytes the MTOC asters are ∼4 fold shorter in MT length, resulting in smaller forces being generated. The motor numbers would then be insufficient to successfully resolve the tug-of-war in mouse oocytes by a simple ∼2-fold changes in MT length, as seen in *Xenopus* oocytes. In contrast, the gradient of immobilized motors result in a comparable spatial distribution of velocity for minus-ended motors with *f*_0_ = 2 pN (S6A Fig) and *f*_0_ = 7 pN (S6B Fig).

Spatial gradients of RanGTP [6, 65] are thought to direct centrosomal MT asters to chromatin during spindle assembly in meiotic extracts [5–7]. However the outcome of our simulations predicts that in meiotic MTOC motility, motor gradients are necessary. Such a gradient could arise from self-organized diffusion and attachment of minus-ended motors on MTs nucleated at the chromatin periphery. In addition, the motors could be immobilized on intracellular organelles, as shown in centrosomal aster centration in *C. elegans* [16]. It remains to be seen which of these specific mechanisms results in the the chromatin centered gradient of anchored motors. Some evidence from experiments in mouse oocytes serves to support our motor gradient model, where pre-NEBD maturing oocytes showed a high-concentration of dynein motors around the nucleus [66]. More recently, evidence of dynein localized at the nuclear periphery fragmenting MTOCs [63], suggests a validation of our model of a chromatin centered gradient of dynein-like motors. Further testing is however required to examine its dynamics.

The role of chromosomes in the centering activity of MTOCs in maturing mouse oocytes during meiotic I spindle assembly had been tested by experimentally removing the nucleus [9, 39]. The MTOCs of such oocytes have a scattered appearance and fail to assemble bipolar spindles. While chromosomes are considered essential for meiotic spindle assembly [67], we hypothesize that they also serve as the primary guidance cue of centripetal MTOC motility, in a manner comparable to other aster cell centering systems [5, 27, 36]. To test this, the spatial organization of MTOCs in enucleated oocytes was measured using published experimental data reported by Schuh and Ellenberg [9]. The image analysis of MTOCs positions from experiment (S7A and S7B Fig) resulted in a radial density distribution around the cell center (S7C Fig). This distribution is comparable to a simulation of randomly localized MTOCs, suggesting the chromatin serves as an important guidance cue for the centripetal motility of MTOCs, and in it’s absence the directional motility is lost. The molecular mechanism which converts the positional information of the chromatin into a gradient of molecular motors, still remains to be understood.

The msd profiles of MTOCs from simulations that were close to chromatin transition from super-diffusive to sub-diffusive motility (S1 Text, S8 Fig), as estimated by *α*, the measure of anomalous diffusion (S8F Fig). This transition results purely from changing the single dynein motor stall force from that reported for bovine brain cytoplasmic dynein (*f*_0_ = 2 pN) [48] to the yeast cytoplasmic dynein (*f*_0_ = 7 pN) [56] value. The MTOC convergence in the 2 pN stall force calculations is slow (S4 Video) as compared to when the motors had a higher stall force (7 pN) (S5 Video) at the same motor density ($N_{m}^{i}=10^{3}$ motors/oocyte). Once the MTOCs reach the center of the motor gradient, they undergo effectively sub-diffusive movement, unable to generate enough force asymmetry to escape. With an additional centrosomal fluorescence label in mouse MTOC motility, the intra-nuclear motility and reorganization of MTOCs could be studied in future. This will also help better understand the subsequent bipolar spindle formation and its connection with chromosome biorientation in meiosis I of mouse oocytes [68, 69].

The sensitivity analysis of the gradient model demonstrates that MT number per aster does not affect velocity and directionality of the asters. On the other hand the model is sensitive to motor density. The motor density in 2D area-density ranging between $N_{m}^{i}=10^{3}$ and 10^4^ motors/oocyte corresponds to a physical density of ≈ 0.2 to 2 motors/*μm*^2^ for an oocyte of radius 40 *μm*. The addition of motor complexes, which cross-link MTs and walk to the minus-ends of the respective MTs, also change the dynamics of MTOC transport. At high densities of motor complexes, the asters coalesce to a few (∼10) clusters and centripetal convergence results. Yet the directionality profiles are qualitatively different from the experimental measures (Fig 6C). While the densities of both immobilized and diffusible motors tested are mean area densities, over-expression of dynein motor proteins could serve as an experimental test of our model predictions.

A limitation of the models described here is that both the phenomenological and mechanistic models assume a 2D circular geometry, while the mouse oocyte is a 3D sphere. So asters finding the cell center in 3D is expected to take longer due to dimensionality in biological search problems [70], and a further parameter optimization would be required. Our effort here serves to reduce the search for ‘scenarios’, i.e. combinations of mechanisms such as motors, MT-dynamic instability, gradients and clustering motors, by optimizing simulations to experimental data. In future, a full 3D model would then only require parameter optimization to a 3D experimental dataset. At the other extreme, a 1D model could have further simplified the geometry based on the radial-symmetry of the system, as has been assumed in the ‘slide and cluster’ model of linear MT filaments in spindle assembly [71]. However this would ignore the orthogonal interactions between MTs of multiple asters, which are only possible in an explicit 2D geometry. Thus our choice of spatial dimensions is driven by an attempt to capture the important qualitative behavior of the system, while keeping the model simple enough for clarity and calculation speed. While a 3D simulations of cellular processes are more ‘complete’, it has been suggested the choice of spatial dimensions should simplify the system to sufficiently capture the spatial dynamics [35, 72]. A further limitation of both the phenomenological RWD and MT-motor model is the availability of only one time-series dataset. In future, additional experiments with mouse oocytes could help test the model predictions. While this model has been developed to test the generality of mechanisms on the mouse MTOC motility, it would be useful in future to explore the relevance of this model to other aster-centering systems such as in *C. elegans* [15], *Xenopus* [27], *Drosophila* [73] and sea urchin [74]. Additional mechanisms such as a contractile actin network has been shown to drive spindle assembly in meiosis I of starfish oocytes [18], but are ignored in this model since in experiments, it does not affect the process [9]. Additionally the potential role of MT-dependent MT nucleation [75] remains to be explored as a self-organized mechanism. Testing alternative cellular geometries and additional mechanisms could in future result in a more complete exploration of MT aster centripetal transport and the physical constraints. This in turn might help us better understand the physical basis of the evolutionary diversity of aster-centering mechanisms.

The models of of MTOC centripetal motility explored in this study, provide insights into several aspects of the early stages of self-organized spindle assembly. For instance, the RWD model demonstrates that for a cell of diameter ≈ 80 *μm*, a simple random-walk strategy of MTOC particles ‘search-and-capture’ at the chromosomes is insufficient within the time-scale of spindle assembly in meiosis (20-30 min) [9], and a long-range bias in motility is essential. The mechanistic model demonstrates that a bias of motors is minimally capable of reproducing the dynamics. MT mass increase which could arise due to increasing MT lengths (stabilization) [5, 6] or number (nucleation) [75], both appear to be insufficient to affect the dynamics of motility. We also find, dynein-like clustering motor complexes, which diffuse and cross-link MTs, at a high density result in an aggregation of MTOC asters to the cell interior. Such a self-organized mechanism however is inefficient in resulting in MTOC capture percentages comparable to experimental values (Table 3) in comparable time-scales (≈ 20 min). Additionally MT density per aster does not affect MTOC centripetal motion but the density of immobilized motors ($N_{m}^{i}$) localized in a gradient does change the dynamics. Such parameters are likely to be adjusted by cells, depending on the specific geometry and time-constraints. The results of this study could be used to predict the nature of MTOC centripetal transport, when the size ratios of asters to cell sizes are also comparable to the mouse oocyte. A more complete picture of the evolutionary constraints on mechanisms driving radial MT arrays to find the center of a cell will however require further quantitative studies from acentrosomal spindle assembly from more organisms, as well as calculations that explore a greater parameter space.

**Fig 7:**
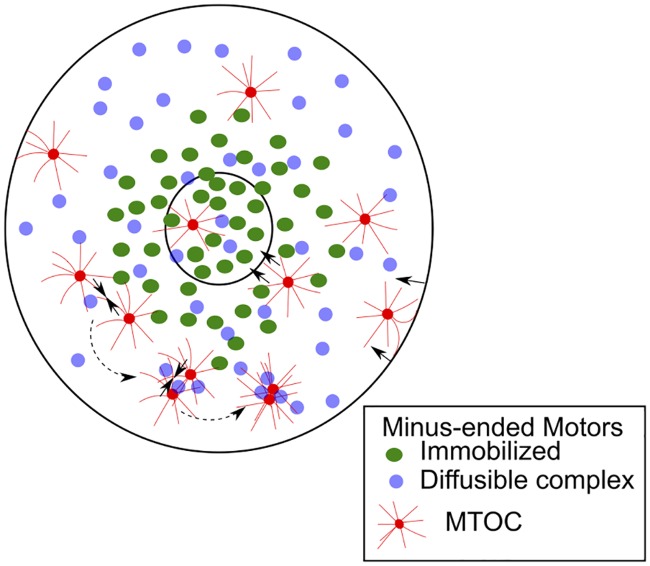
Mechanism of MT centering. An overview of our model of the centering motility of MTOCs (red) in a mouse-oocyte geometry. Net forces (solid arrows) arise from: inward inward pushing due to MT bending at the cell boundary, inward pulling due to a gradient of immobilized motors around the chromatin space (green) and clustering by uniformly distributed diffusible clustering motors (purple). The dotted arrows indicate the process of clustering.

The model presented here predicts the functional form of a drift field in agreement with experimental data of MTOC motility. A mechanistic model of the gradient with immobilized minus-ended motors minimally reproduces experimental dynamics and allows us to test the effect of single molecule characteristics of motors on collective transport statistics. A hybrid model with a gradient of immobilized motors and diffusible clustering dynein-like complexes, combined with cortical pushing (Fig 7), satisfies all experimental measures available. Measuring the localization, density and mobility of dyneins in the mouse oocyte during meiosis I, would be a useful test of the model predictions. While the model is fit to spindle assembly during meiosis I in mouse oocytes, it also predicts the design constraints in terms of MT lengths and the localization, mobility and density of molecular motors when MT radial arrays are required to search space and undergo capture at the cell center.

## Supporting Information

S1 Fig Msd of MTOCs measured in experiment. (A) The msd (*μm*^2^) (blue line) as a function of time is calculated (based on Eq 6) for all available MTOC trajectories from Schuh & Ellenberg [9] and fit to an effective diffusion and drift velocity model (Eq 7). The frequency distribution of the fit parameters (B) effective diffusion coefficient (*D*_*eff*_) (mean 0.006 ± s.d. 0.006 *μm*^2^/*s*) and (C) drift velocity (*v*_*eff*_) (mean 0.008 ± s.d. 0.005 *μm*/*s*) are plotted.
(TIFF)

S2 FigOptimizing gradient parameters.(A) All those parameter sets (k) which fell within the top 15% of the sum of scores ranking are plotted with the component rank for capture time (*R*_*t*_(*k*)) (grey) and directionality (*R*_*χ*_(*k*)) (blue). The parameter sets (k) of the top-ten ranks are listed in S1 Table with the values of *r*_1/2_ and *s* for *ϕ*_*a*_ and *ϕ*_*r*_. For a representative subset of the optimization scheme, the error (*ϵ*) in (B) *χ* and (C) *t*_*c*_ were evaluated keeping the attractive gradient constant ($r_{1/2}^{a}=10$ *μm* and *s*^*a*^ = 1) and varying the repulsive gradient parameters $r_{1/2}^{r}$ (y-axis) and $s_{2}^{r}$ (x-axis). The colorbar indicates the value of *ϵ*.(TIFF)

S3 Fig Fits to distance travelled by MTOC from experiment. The distance travelled of experimentally measured MTOCs were plotted as a function of time in minutes. The plots are sorted based on increasing distance of nucleation (*d*_*n*_) from chromatin. Each profile was fit to the effective model (Eq 11) to obtain a ‘cooperativity parameter’ (*n*).
(TIFF)

S4 Fig MTOC velocity distribution. The experimentally measured frequency distribution of instantaneous velocity (based on XY-trajectories taken from Schuh & Ellenberg [9] and re-analyzed) is fit to a lognormal function. The parameters are the mean *μ* = 8.8 ⋅ 10^−3^ *μm*/*s* and variance *v* = 4.57 ⋅ 10^−5^.
(TIFF)

S5 FigDistance travelled in a motor gradient.MT-motor simulations in presence of a motor gradient (*f*_0_ = 2 pN, $N_{m}^{i}=10^{4}$ motors/oocyte) were used to plot the distance travelled by the MTOCs (z-axis) as a function of time in minutes (x-axis) and nucleation position (y-axis). The plots represent the subset of MTOCs nucleated (A) close to chromatin (0-10 *μm*), (B) in the mid-cell region (10-20 *μm*) and (C) near the cell boundary (20-30 *μm*).(TIFF)

S6 FigSpatial velocity distribution.The mean velocities in *μm*/*s* (y-axis) radially binned as a function of distance from the cell center in *μm* (x-axis) calculated from the MT-motor model in an immobilized motor gradient with motor stall forces and densities: (A) *f*_0_ = 2 pN, $N_{m}^{i}=10^{4}$ motors/oocyte and (B) *f*_0_ = 7 pN and $N_{m}^{i}=10^{3}$ motors/oocyte.(TIFF)

S7 Fig Enucleation and MTOC distributions. (A) Previous data of mouse oocyte enucleation prior to NEBD by Schuh & Ellenberg [9] was used to automatically detect MTOCs (yellow outlines) and their centroids (red asterisk). Scale bar = 10 *μ*m. (B) The 2D coordinates of these experimental MTOC positions were used to (C) compare the radial density distribution of experimentally measured MTOCs (red) with simulated MTOCs (black) that were localized randomly with a uniform density.
(TIFF)

S8 FigMsd as a function of nucleation distance from experiment and simulation.Msd profiles were calculated for the experimentally measured MTOC trajectories and sorted by their nucleation distance (*d*_*n*_) where (A) *d*_*n*_ ≤ 10 *μm*, (B) 10 < *d*_*n*_ ≤ 20 *μm* and (C) 20 < *d*_*n*_ ≤ 30 *μm*. (D) The *D*_*eff*_ and (F) *α* values obtained from fits to simulated msd trajectories are plotted as a function of *d*_*n*_. Experimental profiles (black line) are compared to multiple scenarios in simulation (800 trajectories per scenario) with different stall forces (*f*_0_) and motors per cell ($N_{m}^{i}$). The values are mean ± s.d. The error between simulation and experiment, *ϵ* (colorbar) is plotted for (E) *D*′ and (G) *α* as a function of stall force (*f*_0_) and motor density ($N_{m}^{i}$).(TIFF)

S9 Fig Spatio-temporal trends in apparent diffusion coefficient and anomaly parameter from experiment. The apparent diffusion coefficient (*D*′) and the measure of anomalous diffusion (*α*) were obtained from fitting the anomalous diffusion model (Eq 8) to experimental msd profiles. (A) *D*′ and *α* are plotted as a function of nucleation distance (x-axis) and (B) time duration of the trajectory (x-axis).
(TIFF)

S1 VideoMTOC motility in the absence of a gradient.80 MTOCs (grey) in the oocyte are pushed inwards by the cell boundary (outer blue circle) with $N_{m}^{i}=10^{3}$ immobilized minus-end directed motors (green dots) with *f*_0_ = 7 pN resulting in random MTOC motility. No gradient originates from the chromatin (inner blue circle).(MP4)

S2 VideoMTOC motility in the presence of diffusible minus-ended motor complexes.80 MTOCs (grey) were simulated in cytoplasmic space of the 2D oocyte geometry marked by the outer cell boundary (outer blue circle) and inner chromatin region (blue circle) in the presence of minus-end directed motor complexes (purple dots) initialized in the cytoplasm with density $N_{m}^{c}=10^{3}$ motors/oocyte. These diffusible motors with stall force *f*_0_ = 7 pN, can cross-link MTs and result in clustering by walking towards the minus-ends of neighboring asters that they crosslink.(MP4)

S3 VideoMTOC motility in a gradient of MT dynamic instability.80 MTOCs (grey) were simulated in the 2D oocyte geometry with the outer cell boundary (outer blue circle) and $N_{m}^{i}=10^{3}$ uniformly distributed surface immobilized motors with *f*_0_ = 7 pN. The *f*_*cat*_ and *f*_*res*_ parameters were distributed in a sigmoid gradient (Fig 3G) originating from the center of the chromatin region (inner blue circle).(MP4)

S4 VideoMTOC motility in a gradient of weak motors.80 MTOCs (grey) were simulated in the 2D oocyte geometry with the outer cell boundary (outer blue circle) and $N_{m}^{i}=10^{3}$ surface immobilized motors with *f*_0_ = 2 pN distributed in a sigmoid gradient (Fig 3J) originating from the center of the chromatin region (inner blue circle), and homogeneous dynamic instability.(MP4)

S5 VideoMTOC motility in a gradient of strong motors.80 MTOCs (grey) were simulated in the 2D oocyte geometry with the outer cell boundary (outer blue circle) and $N_{m}^{i}=10^{3}$ surface immobilized motors with *f*_0_ = 7 pN, distributed in a sigmoid gradient (Fig 3J) originating from the center of the chromatin region (inner blue circle).(MP4)

S6 VideoMTOC motility in a gradient of immobilized motors and diffusible motor-complexes.80 MTOCs (grey) were simulated in the 2D oocyte geometry with the outer cell boundary (outer blue circle) and motors which are immobilized at a density of $N_{m}^{i}=10^{3}$ motors/oocyte (green dots) in a sigmoid gradient originating from the center of the chromatin region (inner blue circle). The diffusible minus-end directed motor-complexes with $N_{m}^{c}=10^{4}$ motors/oocyte (purple dots) bind to 2 MTs and walk simultaneously on them, generating a clustering force on the MTOC asters. For both kinds of motors *f*_0_ = 7 pN.(MP4)

S1 TableOptimized RWD gradient parameters.The top ten sum ranks are listed in ascending order, based on the ranks from the directionality and capture time error (*ϵ*(*k*)), with the corresponding parameters of the attractive ($r_{1/2}^{a}$ and *s*^*a*^) and repulsive ($r_{1/2}^{r}$ and *s*^*r*^) gradients (see S2A Fig).(PDF)

S1 Text Msd analysis and anomalous diffusion. (PDF)
