# Supplementary figures and images for: A Motor-Gradient and Clustering Model of the Centripetal Motility of MTOCs in Meiosis I of Mouse Oocytes
Source: PLoS Comput Biol. 2016 Oct 5;12(10):e1005102. doi: 10.1371/journal.pcbi.1005102 (PMC5051731; doi:10.1371/journal.pcbi.1005102)

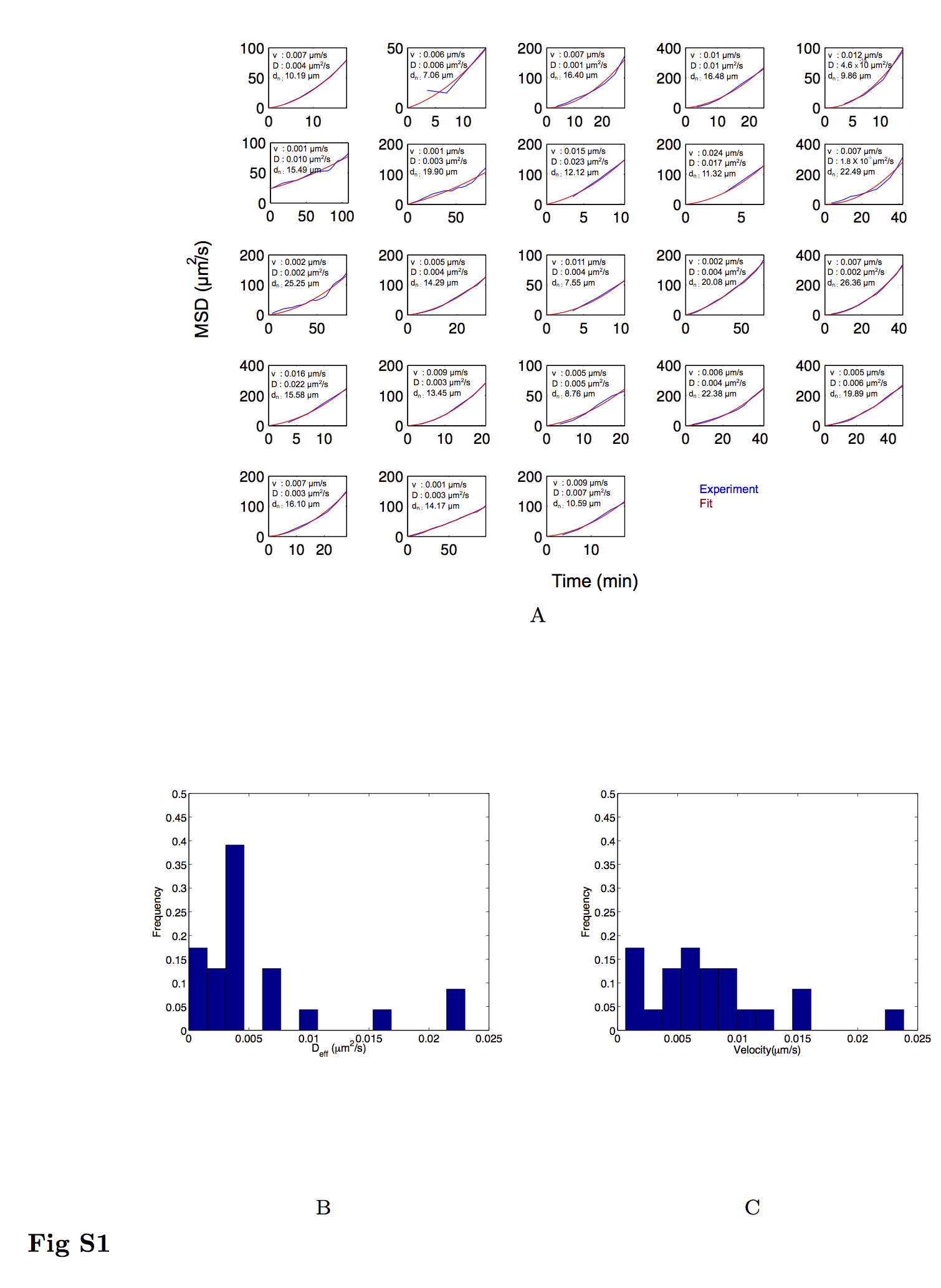

Supplement: S1 Fig — (A) The msd (μm2) (blue line) as a function of time is calculated (based on Eq 6) for all available MTOC trajectories from Schuh & Ellenberg [9] and fit to an effective diffusion and drift velocity model (Eq 7). The frequency distribution of the fit parameters (B) effective diffusion coefficient (Deff) (mean 0.006 ± s.d. 0.006 μm2/s) and (C) drift velocity (veff) (mean 0.008 ± s.d. 0.005 μm/s) are plotted. (TIFF) [file pcbi.1005102.s001.tiff]

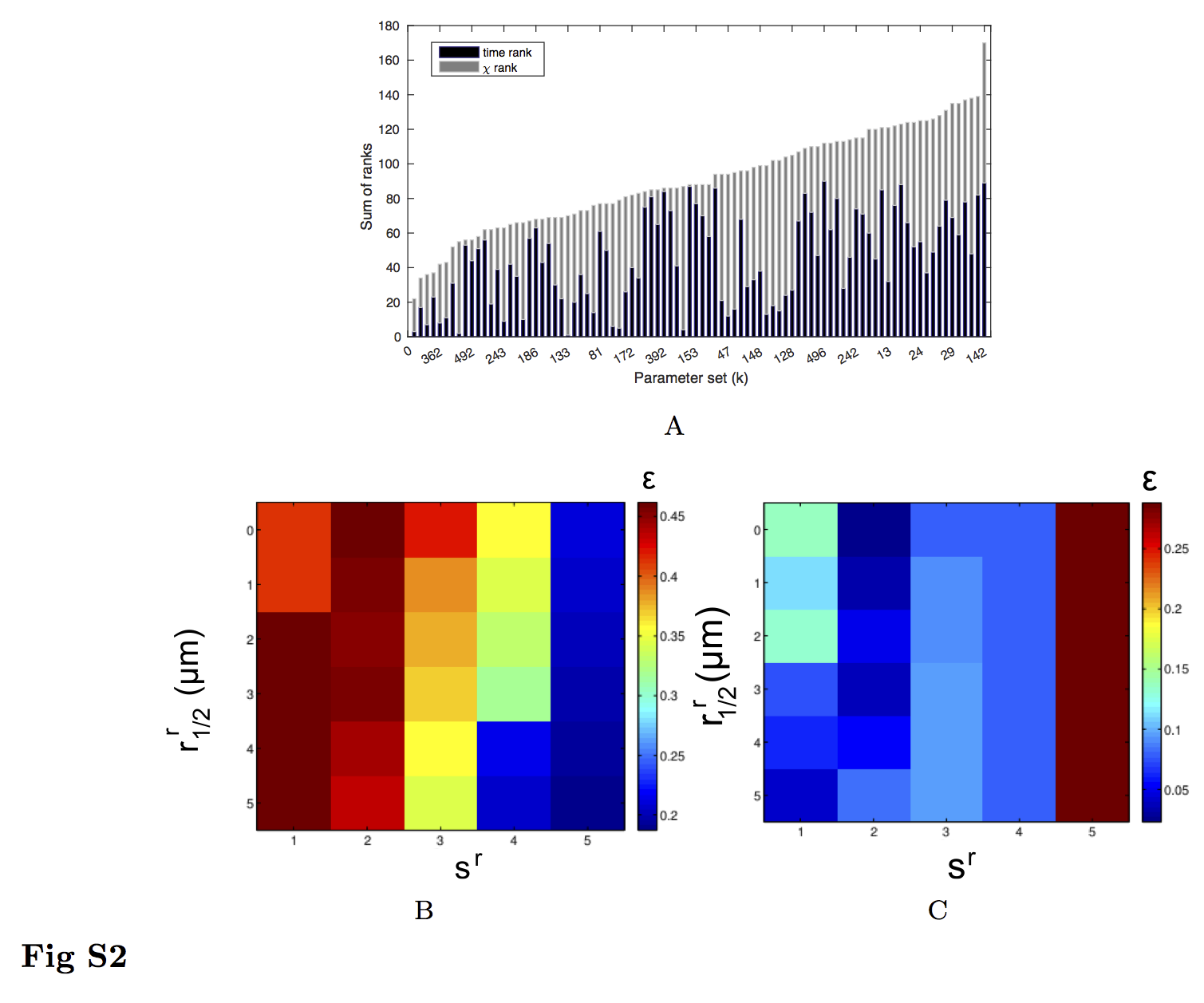

Supplement: S2 Fig — (A) All those parameter sets (k) which fell within the top 15% of the sum of scores ranking are plotted with the component rank for capture time (Rt(k)) (grey) and directionality (Rχ(k)) (blue). The parameter sets (k) of the top-ten ranks are listed in S1 Table with the values of r1/2 and s for ϕa and ϕr. For a representative subset of the optimization scheme, the error (ϵ) in (B) χ and (C) tc were evaluated keeping the attractive gradient constant (r1/2a=10 μm and sa = 1) and varying the repulsive gradient parameters r1/2r (y-axis) and s2r (x-axis). The colorbar indicates the value of ϵ. (TIFF) [file pcbi.1005102.s002.tiff]

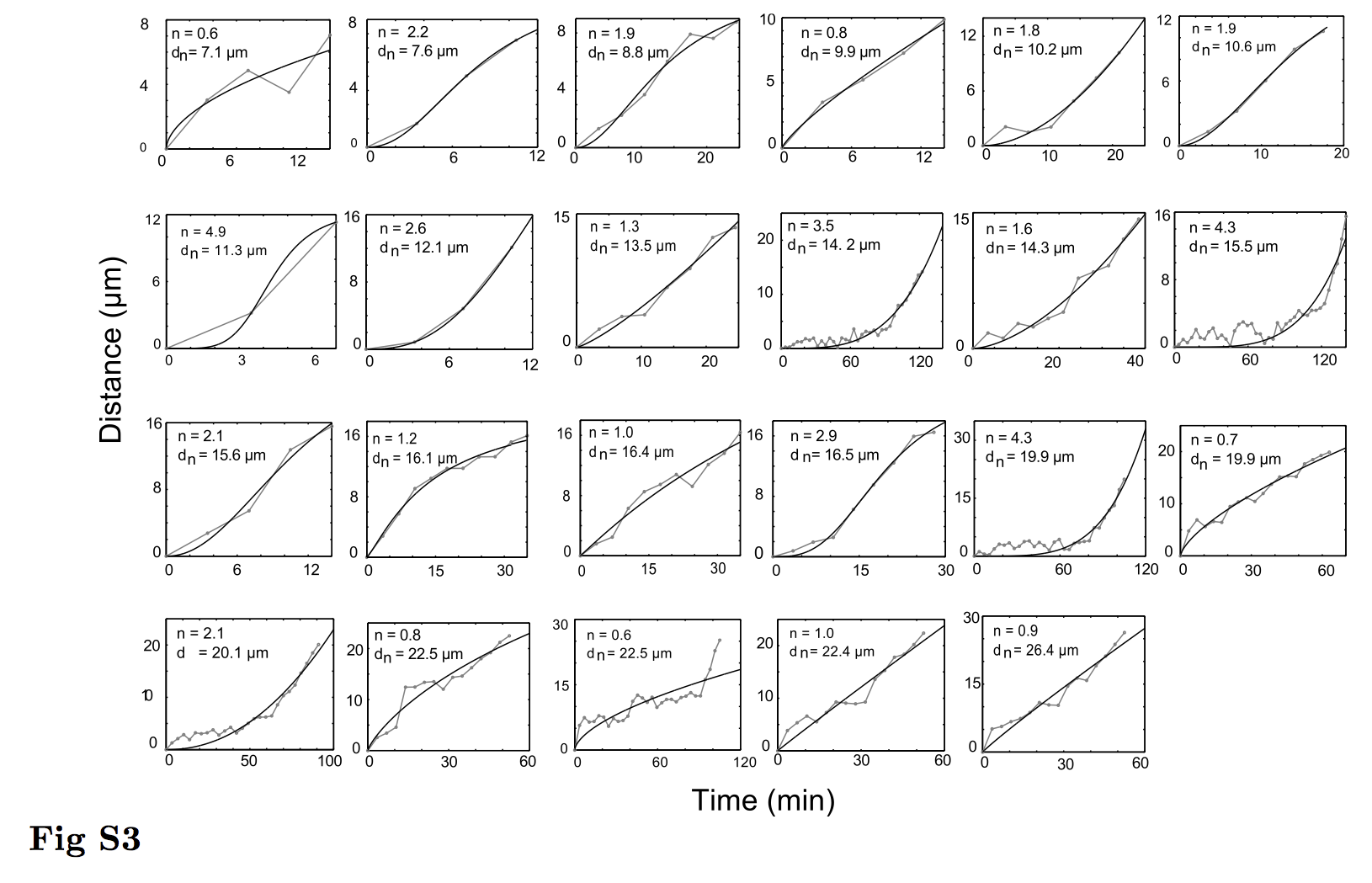

Supplement: S3 Fig — The distance travelled of experimentally measured MTOCs were plotted as a function of time in minutes. The plots are sorted based on increasing distance of nucleation (dn) from chromatin. Each profile was fit to the effective model (Eq 11) to obtain a ‘cooperativity parameter’ (n). (TIFF) [file pcbi.1005102.s003.tiff]

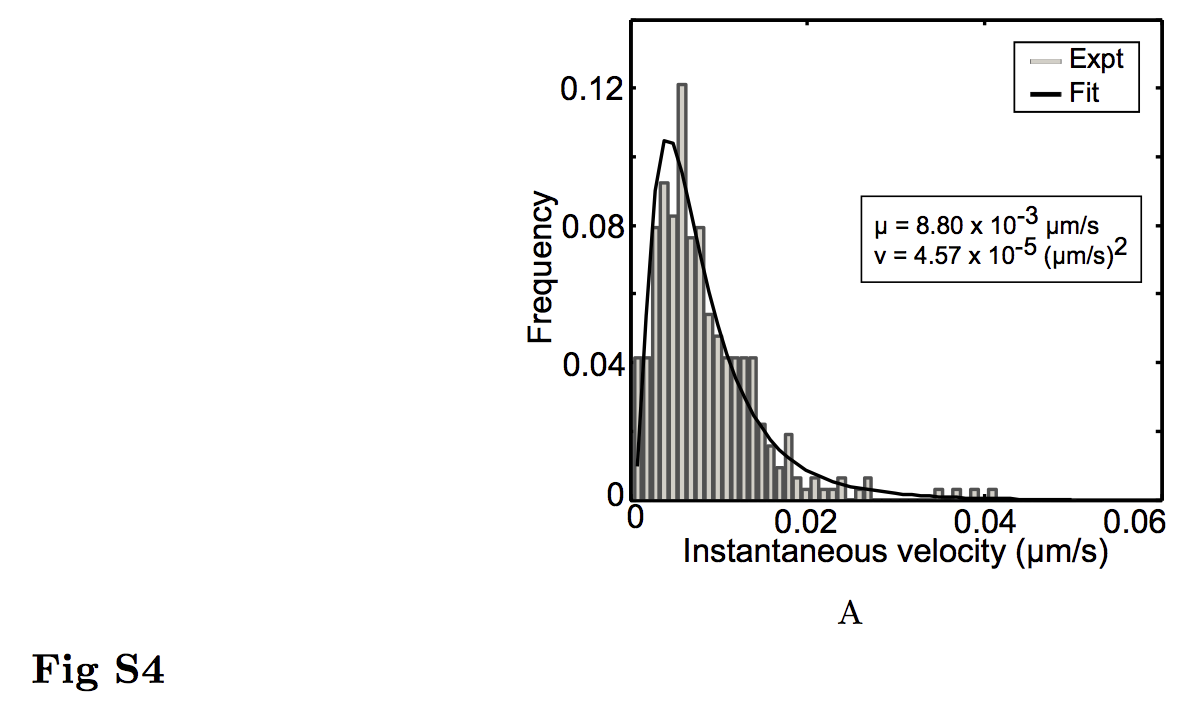

Supplement: S4 Fig — The experimentally measured frequency distribution of instantaneous velocity (based on XY-trajectories taken from Schuh & Ellenberg [9] and re-analyzed) is fit to a lognormal function. The parameters are the mean μ = 8.8 ⋅ 10−3 μm/s and variance v = 4.57 ⋅ 10−5. (TIFF) [file pcbi.1005102.s004.tiff]

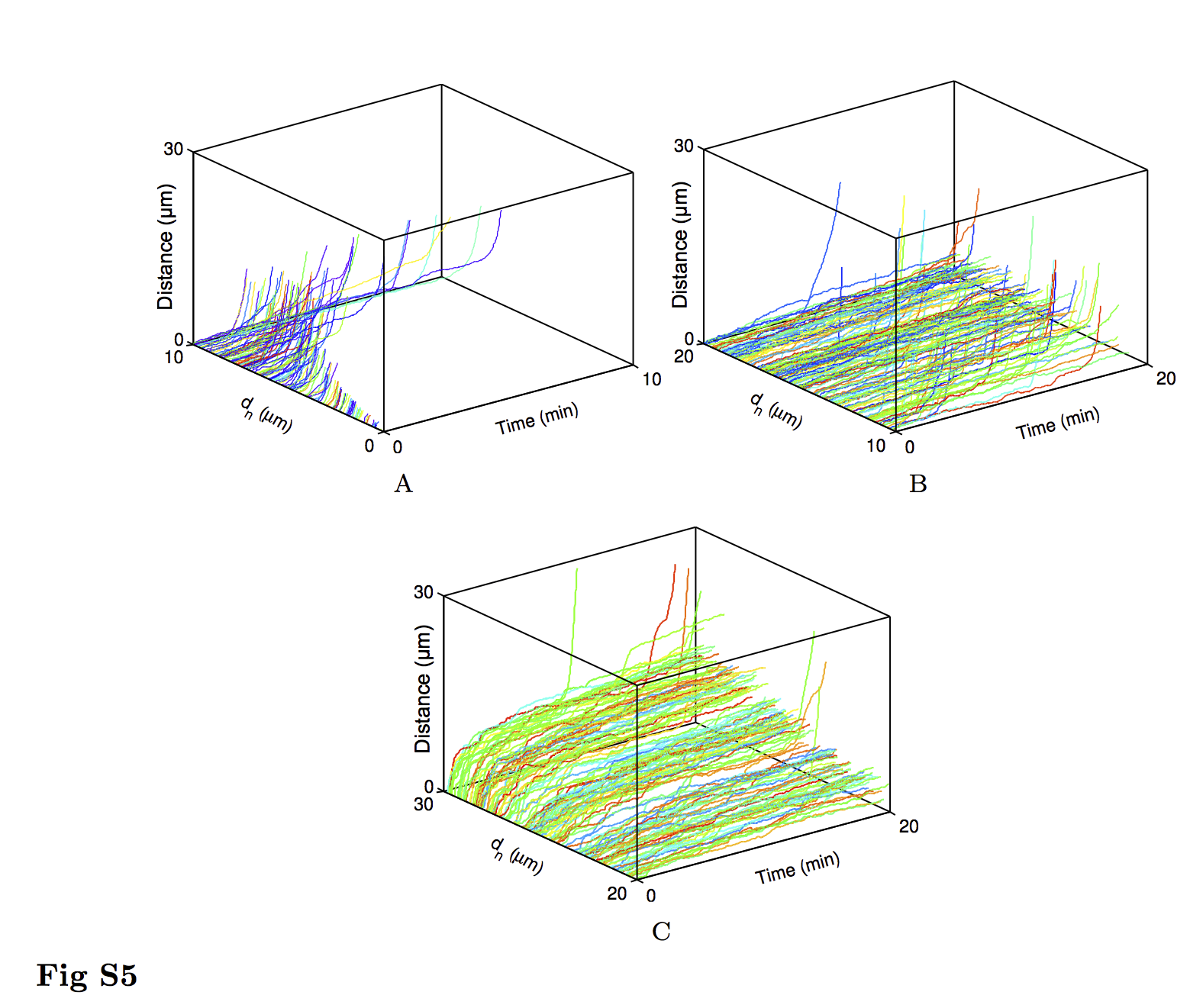

Supplement: S5 Fig — MT-motor simulations in presence of a motor gradient (f0 = 2 pN, Nmi=104 motors/oocyte) were used to plot the distance travelled by the MTOCs (z-axis) as a function of time in minutes (x-axis) and nucleation position (y-axis). The plots represent the subset of MTOCs nucleated (A) close to chromatin (0-10 μm), (B) in the mid-cell region (10-20 μm) and (C) near the cell boundary (20-30 μm). (TIFF) [file pcbi.1005102.s005.tiff]

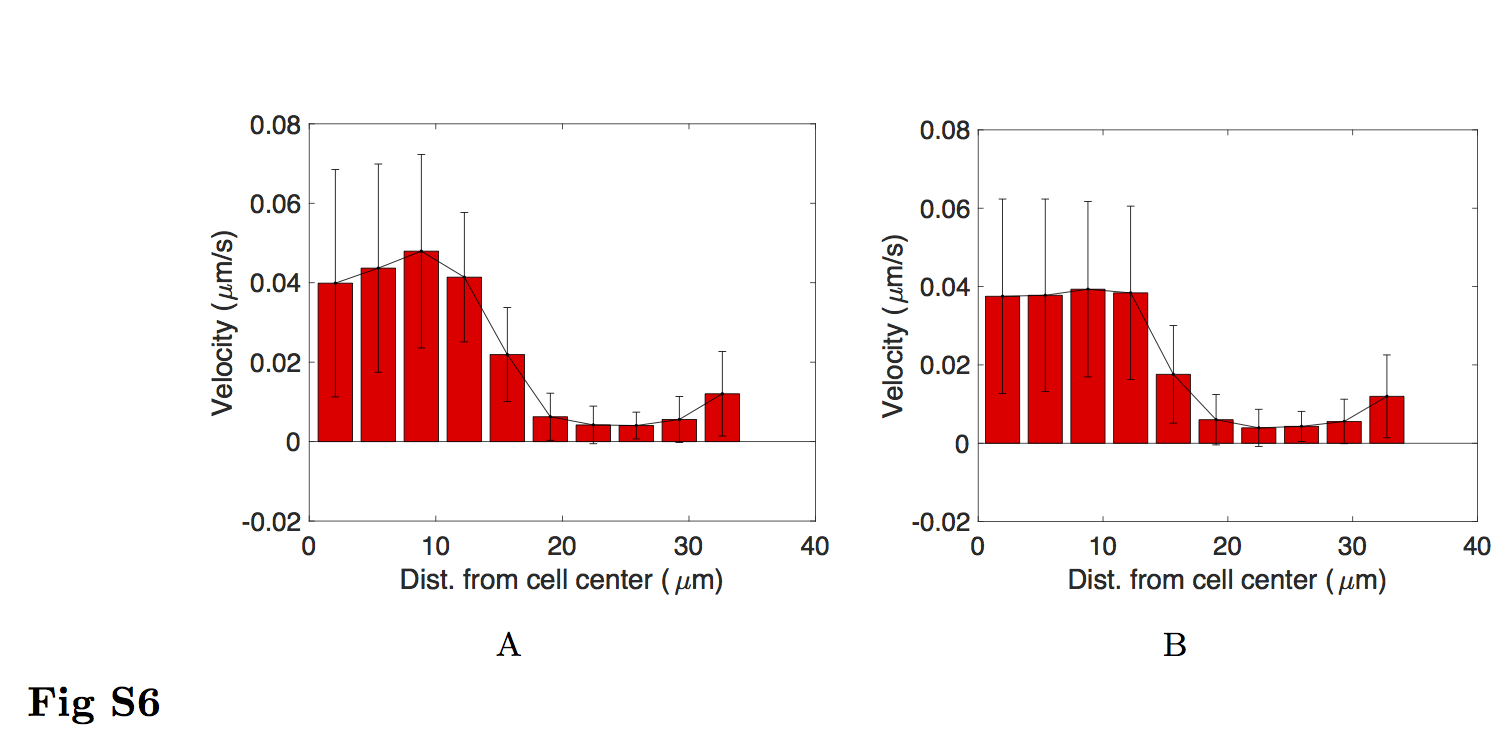

Supplement: S6 Fig — The mean velocities in μm/s (y-axis) radially binned as a function of distance from the cell center in μm (x-axis) calculated from the MT-motor model in an immobilized motor gradient with motor stall forces and densities: (A) f0 = 2 pN, Nmi=104 motors/oocyte and (B) f0 = 7 pN and Nmi=103 motors/oocyte. (TIFF) [file pcbi.1005102.s006.tiff]

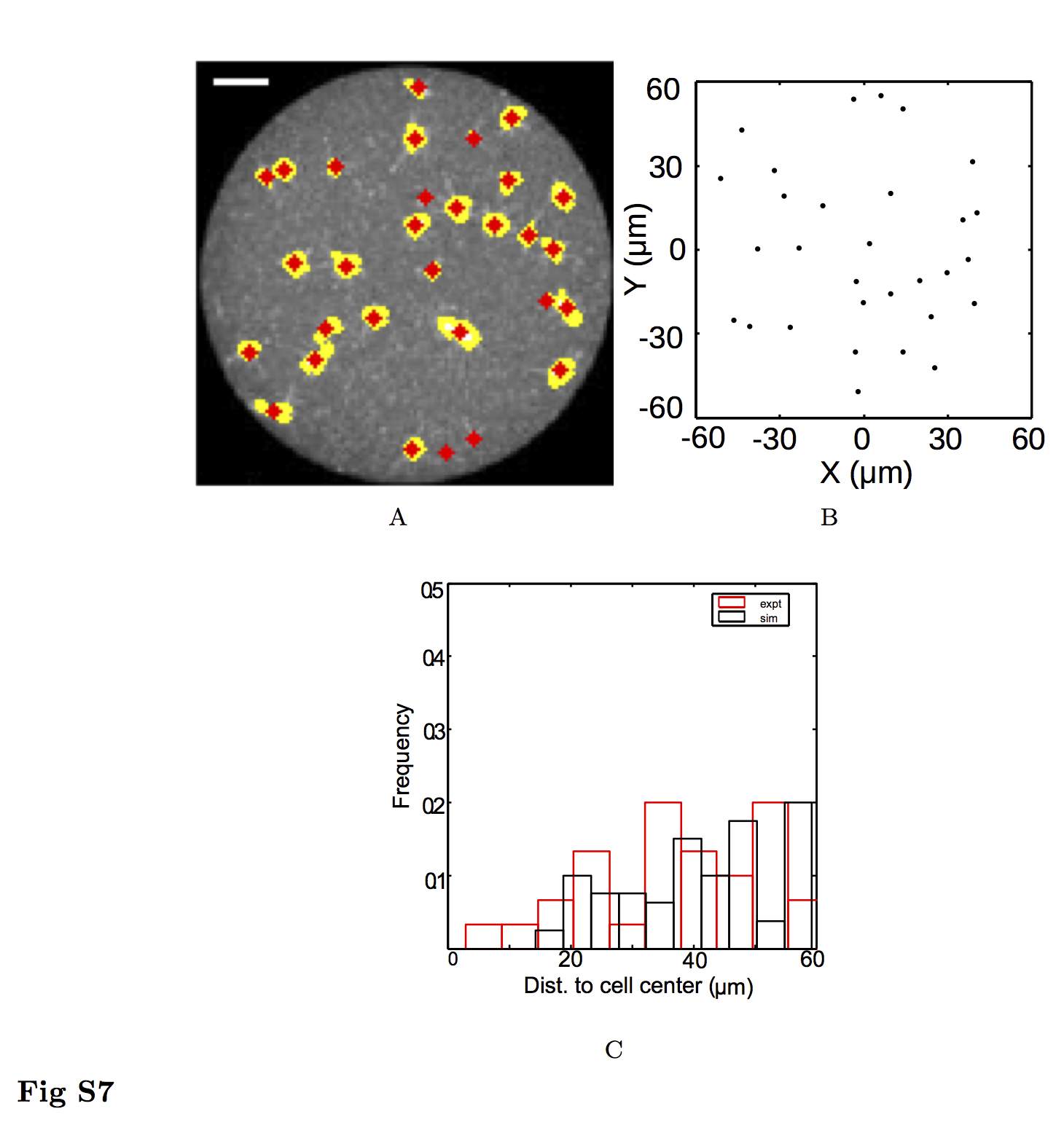

Supplement: S7 Fig — (A) Previous data of mouse oocyte enucleation prior to NEBD by Schuh & Ellenberg [9] was used to automatically detect MTOCs (yellow outlines) and their centroids (red asterisk). Scale bar = 10 μm. (B) The 2D coordinates of these experimental MTOC positions were used to (C) compare the radial density distribution of experimentally measured MTOCs (red) with simulated MTOCs (black) that were localized randomly with a uniform density. (TIFF) [file pcbi.1005102.s007.tiff]

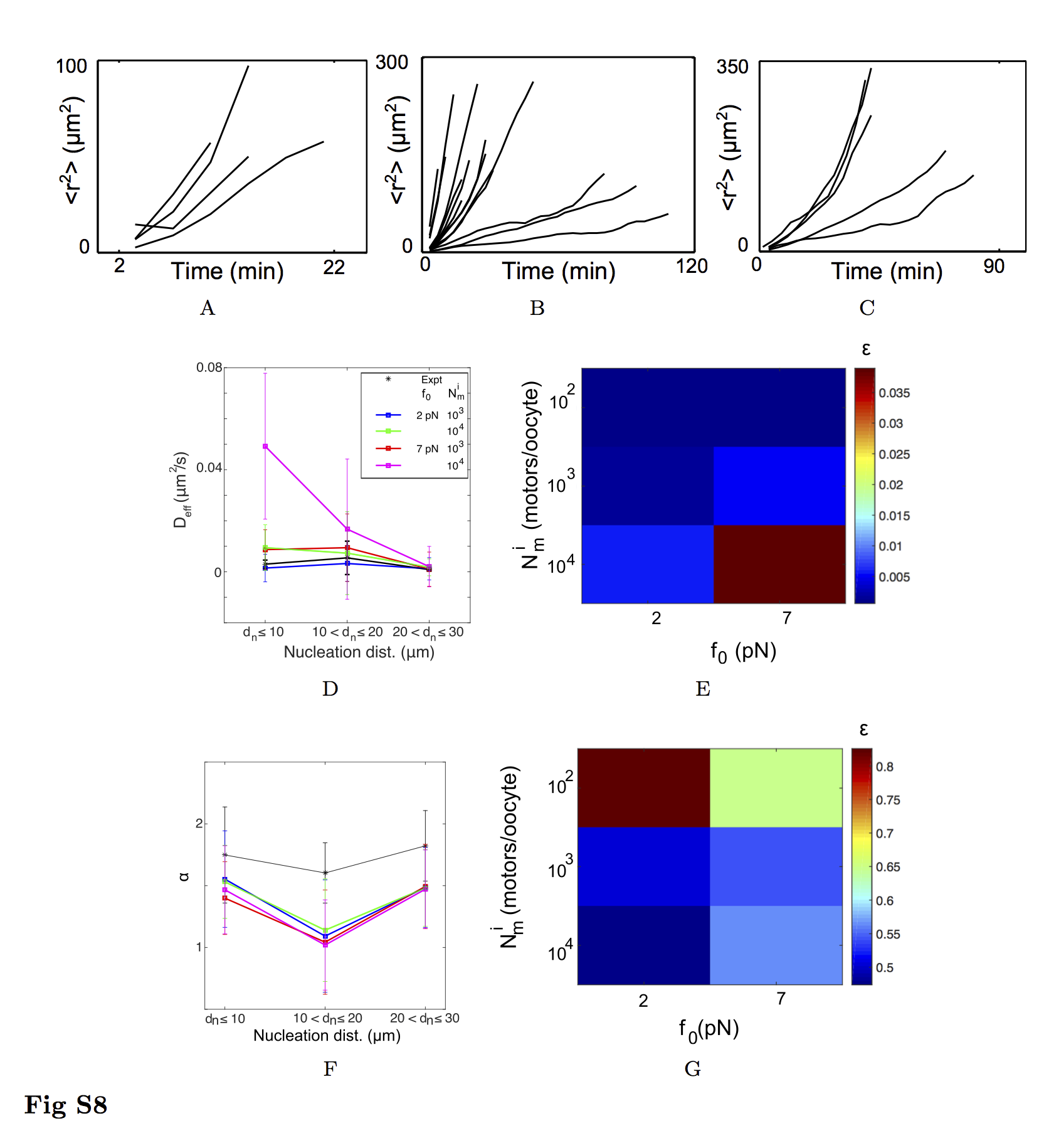

Supplement: S8 Fig — Msd profiles were calculated for the experimentally measured MTOC trajectories and sorted by their nucleation distance (dn) where (A) dn ≤ 10 μm, (B) 10 < dn ≤ 20 μm and (C) 20 < dn ≤ 30 μm. (D) The Deff and (F) α values obtained from fits to simulated msd trajectories are plotted as a function of dn. Experimental profiles (black line) are compared to multiple scenarios in simulation (800 trajectories per scenario) with different stall forces (f0) and motors per cell (Nmi). The values are mean ± s.d. The error between simulation and experiment, ϵ (colorbar) is plotted for (E) D′ and (G) α as a function of stall force (f0) and motor density (Nmi). (TIFF) [file pcbi.1005102.s008.tiff]

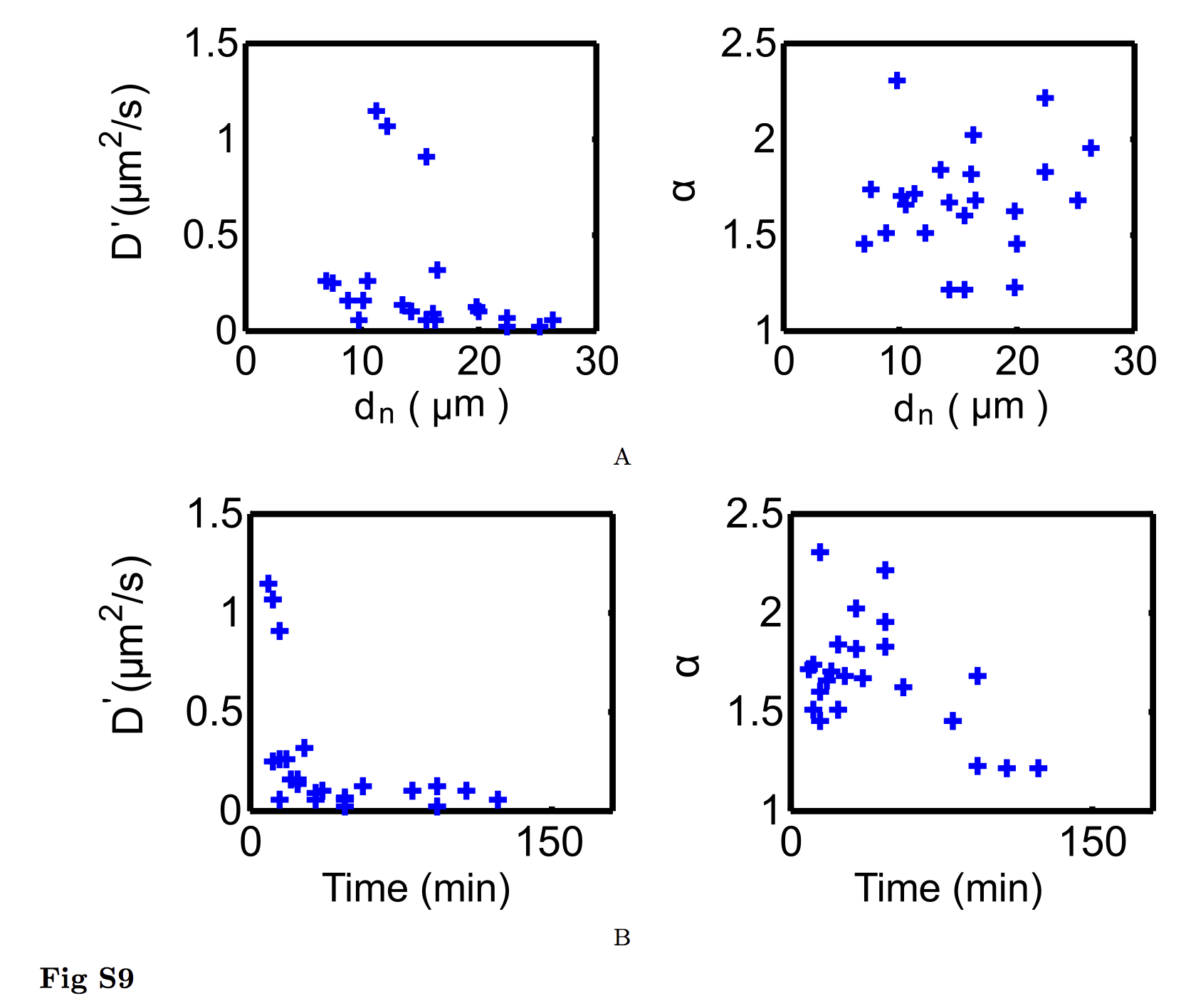

Supplement: S9 Fig — The apparent diffusion coefficient (D′) and the measure of anomalous diffusion (α) were obtained from fitting the anomalous diffusion model (Eq 8) to experimental msd profiles. (A) D′ and α are plotted as a function of nucleation distance (x-axis) and (B) time duration of the trajectory (x-axis). (TIFF) [file pcbi.1005102.s009.tiff]
