# Supplementary material for: A Motor-Gradient and Clustering Model of the Centripetal Motility of MTOCs in Meiosis I of Mouse Oocytes
Source: PLoS Comput Biol. 2016 Oct 5;12(10):e1005102. doi: 10.1371/journal.pcbi.1005102 (PMC5051731; doi:10.1371/journal.pcbi.1005102)
Supplement: S1 Table — The top ten sum ranks are listed in ascending order, based on the ranks from the directionality and capture time error (ϵ(k)), with the corresponding parameters of the attractive (r1/2a and sa) and repulsive (r1/2r and sr) gradients (see S2A Fig). (PDF) [file pcbi.1005102.s016.pdf]

Table S1

S1 Table. Optimized RWD gradient parameters: The top ten sum ranks are listed in ascending order, based on the ranks from the directionality and capture time error ($\epsilon(k)$), with the corresponding parameters of the attractive ($r^a_{1/2}$ and s^a) and repulsive ($r^r_{1/2}$ and s^r) gradients (see Fig S2(A)).

**Parameter set (k)**	**Sum of ranks ($R_s(k)$)**	$r^a_{1/2}$	s^a	$r^r_{1/2}$	s^r
482	22	10	1	0	2
487	34	10	1	1	2
377	36	8	1	3	2
497	37	10	1	3	2
362	42	8	1	0	2
372	43	8	1	2	2
506	52	10	1	5	1
267	55	6	1	5	2
363	56	8	1	0	3
492	56	10	1	2	2
