# Supplementary material for: A Motor-Gradient and Clustering Model of the Centripetal Motility of MTOCs in Meiosis I of Mouse Oocytes
Source: PLoS Comput Biol. 2016 Oct 5;12(10):e1005102. doi: 10.1371/journal.pcbi.1005102 (PMC5051731; doi:10.1371/journal.pcbi.1005102)

Supporting Information

Supporting Text

Msd analysis and anomalous diffusion

In previous work, we have shown that MT-transport results in a tug-of-war of motors when radial MT arrays are transported by a sheet of immobilized molecular motors. Such a tug-of-war has been quantified by evaluating the nature of the msd profiles, and qualitatively distinct msd profiles can be identified based on the fit of msd to the model of anomalous diffusion (Equation 8) where D' is the apparent diffusion coefficient and α the anomaly parameter. Experimentally measured msd plots of MTOCs appear to be directed in their motion, near the cell center (Fig S8A), in the mid-cell region (Fig S8B) and at the cell periphery (Fig S8C). The radial distance of nucleation does not change experimental $D^{'}$ significantly (Fig S8D, Fig S9A) and simulations with low-densities of both motor types ($f_0 = 2$ and 7 pN) reproduce this trend. The experimental anomalous diffusion exponent α is consistently > 1, indicating of anomalous super-diffusion, and is also invariant with nucleation distance (Fig S8F, Fig S9(A)). The short-lived trajectories appear to have higher apparent diffusion coefficients, as compared to long-lived ones and the α value decreases with increasing time for which the trajectory was followed (Fig S9(B)).

The deviation (ϵ) between simulations and experiments of the averaged $D^{'}$ (Fig S8E) and α (Fig S8G), suggest a gradient of weak ($f_0 = 2$ pN) motors with a density of 10^4 motors/oocyte are qualitatively closest to experimental data. The α from the motor-gradient model is consistently lower than experiment. This deviation between simulation and experiment in the mid-cell region is also observed in the χ measure in the mid-cell region, where the experimental value is higher than simulation (Fig 4). This would suggest potentially short-range directional motions in the mid-cell region, might cause this effect.

Fig S1

Msd of MTOCs measured in experiment. (A) The msd (μm^2) (blue line) as a function of time is calculated (based on Equation 6) for all available MTOC trajectories from Schuh & Ellenberg [9] and fit to an effective diffusion and drift velocity model (Equation 7). The frequency distribution of the fit parameters (B) effective diffusion coefficient (D_{eff}) (mean 0.006± s.d. 0.006 $\mu m^2/s$) and (C) drift velocity (v_{eff}) (mean 0.008± s.d. 0.005 $\mu m/s$) are plotted.

Fig S2

Optimizing gradient parameters. (A) All those parameter sets (k) which fell within the top 15% of the sum of scores ranking are plotted with the component rank for capture time ($R_t(k)$) (grey) and directionality ($R_\chi(k)$) (blue). The parameter sets (k) of the top-ten ranks are listed in S1 Table with the values of $r_{1/2}$ and s for ϕ_a and ϕ_r. For a representative subset of the optimization scheme, the error (ϵ) in (B) χ and (C) t_c were evaluated keeping the attractive gradient constant ($r^a_{1/2} = 10$ μm and $s^a = 1$) and varying the repulsive gradient parameters $r^r_{1/2}$ (y-axis) and s^r_2 (x-axis). The colorbar indicates the value of ϵ.

Fig S3

Fits to distance travelled by MTOC from experiment. The distance travelled of experimentally measured MTOCs were plotted as a function of time in minutes. The plots are sorted based on increasing distance of nucleation (d_n) from chromatin. Each profile was fit to the effective model (Equation 11) to obtain a 'cooperativity parameter' (n).

Fig S4

MTOC velocity distribution. The experimentally measured frequency distribution of instantaneous velocity (based on XY-trajectories taken from Schuh & Ellenberg [9] and re-analyzed) is fit to a lognormal function. The parameters are the mean $\mu = 8.8 \cdot 10^{-3}$ $\mu m/s$ and variance $v = 4.57 \cdot 10^{-5}$.

Fig S5

Distance travelled in a motor gradient. MT-motor simulations in presence of a motor gradient ($f_0 = 2$ pN, $N^i_m = 10^4$ motors/oocyte) were used to plot the distance travelled by the MTOCs (z-axis) as a function of time in minutes (x-axis) and nucleation position (y-axis). The plots represent the subset of MTOCs nucleated (A) close to chromatin (0-10 μm), (B) in the mid-cell region (10-20 μm) and (C) near the cell boundary (20-30 μm).

Fig S6

Spatial velocity distribution. The mean velocities in $\mu m/s$ (y-axis) radially binned as a function of distance from the cell center in μm (x-axis) calculated from the MT-motor model in an immobilized motor gradient with motor stall forces and densities: (A) $f_0 = 2$ pN, $N^i_m = 10^4$ motors/oocyte and (B) $f_0 = 7$ pN and $N^i_m = 10^3$ motors/oocyte.

Fig S7

Enucleation and MTOC distributions. (A) Previous data of mouse oocyte enucleation prior to NEBD by Schuh & Ellenberg [9] was used to automatically detect MTOCs (yellow outlines) and their centroids (red asterisk). Scale bar = 10 μm. (B) The 2D coordinates of these experimental MTOC positions were used to (C) compare the radial density

distribution of experimentally measured MTOCs (red) with simulated MTOCs (black) that were localized randomly with a uniform density.

Fig S8

Msd as a function of nucleation distance from experiment and simulation. Msd profiles were calculated for the experimentally measured MTOC trajectories and sorted by their nucleation distance (d_n) where (A) $d_n \leq 10\ \mu m$, (B) $10 < d_n \leq 20\ \mu m$ and (C) $20 < d_n \leq 30\ \mu m$. (D) The D_{eff} and (F) α values obtained from fits to simulated msd trajectories are plotted as a function of d_n. Experimental profiles (black line) are compared to multiple scenarios in simulation (800 trajectories per scenario) with different stall forces (f_0) and motors per cell (N_m^i). The values are mean±s.d. The error between simulation and experiment, ϵ (colorbar) is plotted for (E) $D^{'}$ and (G) α as a function of stall force (f_0) and motor density (N_m^i).

Fig S9

Spatio-temporal trends in apparent diffusion coefficient and anomaly parameter from experiment. The apparent diffusion coefficient ($D^{'}$) and the measure of anomalous diffusion (α) were obtained from fitting the anomalous diffusion model (Equation 8) to experimental msd profiles. (A) D' and α are plotted as a function of nucleation distance (x-axis) and (B) time duration of the trajectory (x-axis).

S1 Video

MTOC motility in the absence of a gradient. 80 MTOCs (grey) in the oocyte are pushed inwards by the cell boundary (outer blue circle) with $N_m^i = 10^3$ immobilized minus-end directed motors (green dots) with $f_0 = 7$ pN resulting in random MTOC motility. No gradient originates from the chromatin (inner blue circle).

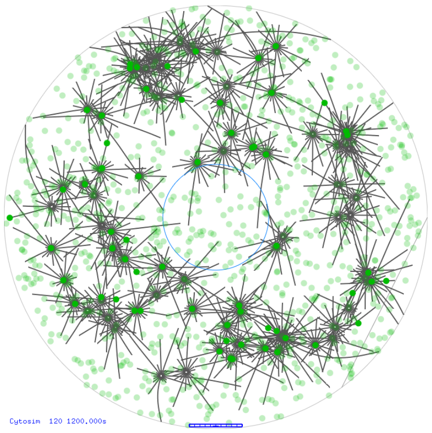

S2 Video

MTOC motility in the presence of diffusible minus-ended motor complexes. 80 MTOCs (grey) were simulated in cytoplasmic space of the 2D oocyte geometry marked by the outer cell boundary (outer blue circle) and inner chromatin region (blue circle) in the presence of minus-end directed motor complexes (purple dots) initialized in the cytoplasm with density $N_m^c = 10^3$ motors/oocyte. These diffusible motors with stall force $f_0 = 7$ pN, can cross-link MTs and result in clustering by walking towards the minus-ends of neighboring asters that they crosslink.

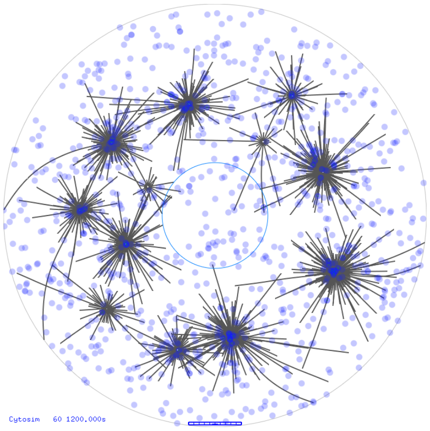

S3 Video

MTOC motility in a gradient of MT dynamic instability. 80 MTOCs (grey) were simulated in the 2D oocyte geometry with the outer cell boundary (outer blue circle) and $N_m^i = 10^3$ uniformly distributed surface immobilized motors with $f_0 = 7$ pN. The f_{cat} and f_{res} parameters were distributed in a sigmoid gradient (Fig 3G) originating from the center of the chromatin region (inner blue circle).

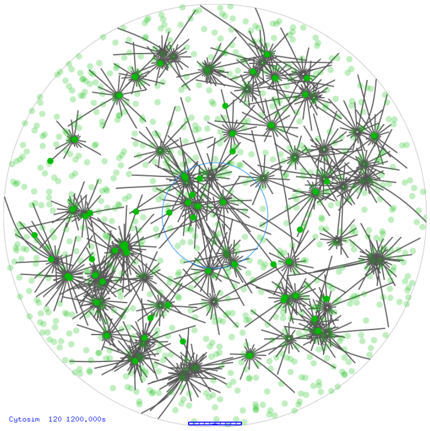

S4 Video

MTOC motility in a gradient of weak motors. 80 MTOCs (grey) were simulated in the 2D oocyte geometry with the outer cell boundary (outer blue circle) and $N_m^i = 10^3$ surface immobilized motors with $f_0 = 2$ pN distributed in a sigmoid gradient (Fig 3J) originating from the center of the chromatin region (inner blue circle), and homogeneous dynamic instability.

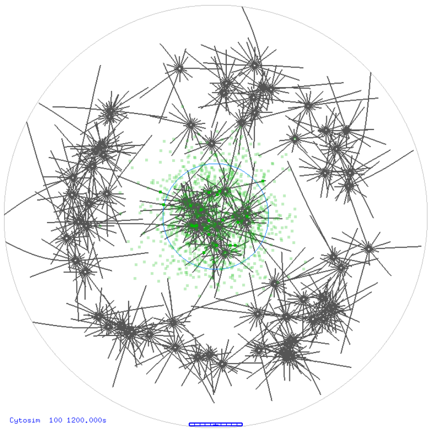

S5 Video

MTOC motility in a gradient of strong motors: 80 MTOCs (grey) were simulated in the 2D oocyte geometry with the outer cell boundary (outer blue circle) and $N_m^i = 10^3$ surface immobilized motors with $f_0 = 7$ pN, distributed in a sigmoid gradient (Fig 3J) originating from the center of the chromatin region (inner blue circle).

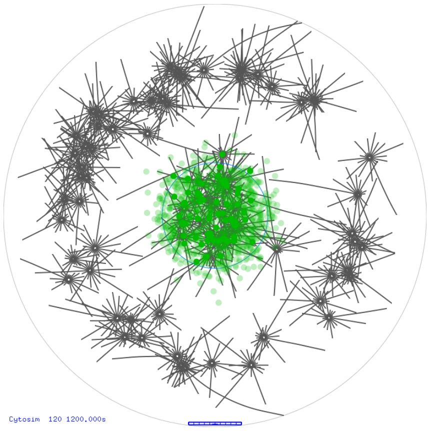

S6 Video

MTOC motility in a gradient of immobilized motors and diffusible motor-complexes: 80 MTOCs (grey) were simulated in the 2D oocyte geometry with the outer cell boundary (outer blue circle) and motors which are immobilized at a density of $N_m^i = 10^3$ motors/oocyte (green dots) in a sigmoid gradient originating from the center of the chromatin region (inner blue circle). The diffusible minus-end directed motor-complexes with $N_m^c = 10^4$ motors/oocyte (purple dots) bind to 2 MTs and walk simultaneously on them, generating a clustering force on the MTOC asters. For both kinds of motors $f_0 = 7$ pN.

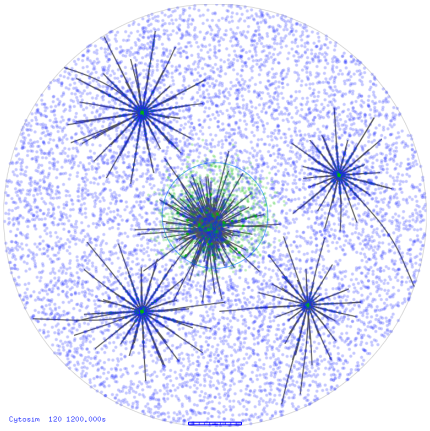

Supplement: S1 Text — (PDF) [file pcbi.1005102.s017.pdf]
